# Common protein sequence signatures associate with *Sclerotinia borealis* lifestyle and secretion in fungal pathogens of the *Sclerotiniaceae*

**DOI:** 10.3389/fpls.2015.00776

**Published:** 2015-09-24

**Authors:** Thomas Badet, Rémi Peyraud, Sylvain Raffaele

**Affiliations:** ^1^Laboratoire des Interactions Plantes-Microorganismes, Institut National de la Recherche Agronomique, UMR441Castanet-Tolosan, France; ^2^Laboratoire des Interactions Plantes-Microorganismes, Centre National de la Recherche Scientifique, UMR2594Castanet-Tolosan, France

**Keywords:** secretome, *Sclerotinia*, psychrophily, effector candidates, amino acid usage, intrinsic disorder, antifreeze protein, lytic polysaccharide monooxygenase

## Abstract

Fungal plant pathogens produce secreted proteins adapted to function outside fungal cells to facilitate colonization of their hosts. In many cases such as for fungi from the *Sclerotiniaceae* family the repertoire and function of secreted proteins remains elusive. In the *Sclerotiniaceae*, whereas *Sclerotinia sclerotiorum* and *Botrytis cinerea* are cosmopolitan broad host-range plant pathogens, *Sclerotinia borealis* has a psychrophilic lifestyle with a low optimal growth temperature, a narrow host range and geographic distribution. To spread successfully, *S. borealis* must synthesize proteins adapted to function in its specific environment. The search for signatures of adaptation to *S. borealis* lifestyle may therefore help revealing proteins critical for colonization of the environment by *Sclerotiniaceae* fungi. Here, we analyzed amino acids usage and intrinsic protein disorder in alignments of groups of orthologous proteins from the three *Sclerotiniaceae* species. We found that enrichment in Thr, depletion in Glu and Lys, and low disorder frequency in hot loops are significantly associated with *S. borealis* proteins. We designed an index to report bias in these properties and found that high index proteins were enriched among secreted proteins in the three *Sclerotiniaceae* fungi. High index proteins were also enriched in function associated with plant colonization in *S. borealis*, and in *in planta*-induced genes in *S. sclerotiorum*. We highlight a novel putative antifreeze protein and a novel putative lytic polysaccharide monooxygenase identified through our pipeline as candidate proteins involved in colonization of the environment. Our findings suggest that similar protein signatures associate with *S. borealis* lifestyle and with secretion in the *Sclerotiniaceae*. These signatures may be useful for identifying proteins of interest as targets for the management of plant diseases.

## Introduction

**Figure 1:**
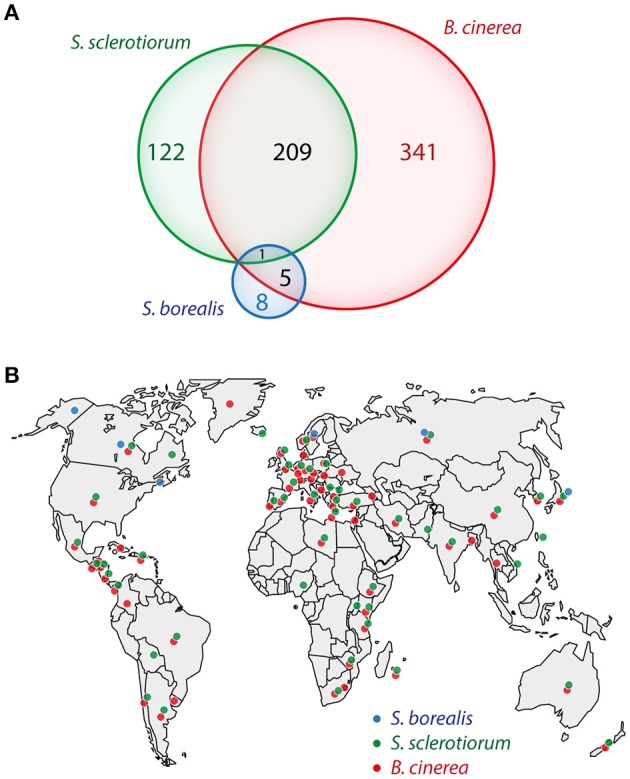
***Sclerotinia borealis* colonizes different niches than its close relatives *S. sclerotiorum* and *Botrytis cinerea***. Number of host plant genera **(A)** and geographic distribution **(B)** of the three fungal species according to the USDA Systematic Mycology and Microbiology Laboratory Fungus-Host Database (Farr and Rossman, 2015).

Fungi from the *Sclerotiniaceae* family include several devastating plant pathogens with a broad host range. Among those are *Botrytis cinerea*, the causal agent of gray rot, and *Sclerotinia sclerotiorum*, causal agent of white and stem rot, each able to infect several hundreds of plant genera and causing multi-billion dollar losses in agriculture every year (Figure 1A) (Bolton et al., 2006; Dean et al., 2012). The geographic distribution of these two fungi is also remarkably broad since they have been reported across five continents (Figure 1B). Sequencing of the genome of *B. cinerea* and *S. sclerotiorum* (Amselem et al., 2011) opened the way to systematic searches for the molecular bases of pathogenicity in these fungi (Guyon et al., 2014; Heard et al., 2015). However, the repertoire of molecules contributing to the ability of plant pathogenic fungi, such as fungi from the *Sclerotiniaceae* family, to colonize a wide range of hosts and environments remains elusive.

Fungal pathogens secrete diverse sets of degrading enzymes and toxins to facilitate colonization of their hosts (Möbius and Hertweck, 2009; Kubicek et al., 2014). In addition, fungal pathogens use molecules designated as effectors to manipulate host cells and achieve successful infection. Their activities include the inactivation of plant defenses, interference with plant hormone balance, or dismantling of the plant cell. However, effectors may also trigger specific plant defense responses, leading to plant resistance, when recognized directly or indirectly by the plant immune system (Jones and Dangl, 2006). Typical effectors are small secreted proteins, but secondary metabolites and small RNAs can also play the role of effectors (Schardl et al., 2013; Weiberg et al., 2013). Although a subset of bacterial and oomycete protein effectors can be identified based on conserved N-terminal targeting signals and other sequence signatures (Schornack et al., 2009; McDermott et al., 2011; Meyer et al., 2013), this is not the case in fungi. Effector detection in fungal pathogens relies largely on specific host responses revealing effector recognition, and bioinformatics approaches based on whole genome sequences and deduced protein repertoires remain challenging (Sperschneider et al., 2015). Genes involved in host-parasite interactions such as pathogen effectors are often subject to strong balancing or directional selection. For example, oomycete effectors commonly evolve rapidly, and natural selection can maintain many different alleles in a population (Raffaele et al., 2010; Oliva et al., 2015). Therefore, signatures of positive selection are frequent in effector genes and this property has been used to identify novel effector candidates (Wicker et al., 2013; Rech et al., 2014; Sperschneider et al., 2014). However, most of our understanding of the molecular evolution of effector genes and genes involved in colonization of the environment comes from studies of the pairwise co-evolution of a given pathogen with a single host plant. By contrast, fungal pathogens in the *Sclerotiniaceae* interact with a wide range of hosts in multiple environmental conditions and should therefore be considered as evolving under “diffuse” (or “generalized”) interactions (Juenger and Bergelson, 1998). In the Ascomycete genus *Metarhizium*, signatures of positive selection were observed less frequently in the genome of fungal pathogens under diffuse co-evolution compared to *Metarhizium acridum* evolving under pairwise co-evolution (Hu et al., 2014). It is thus expected that in the *Sclerotiniaceae*, some genes important for colonization of environment, including fungal effectors involved in diffuse interactions, may escape detection by positive selection analysis, and additional detection criteria would be useful.

Compared to *B. cinerea* and *S. sclerotiorum*, the snow mold pathogen *Sclerotinia borealis* colonizes a reduced range of environments. Indeed, according to the Fungus-Host database of the U.S. Department of Agriculture (Farr and Rossman, 2015), *S. borealis* has been reported to infect 14 plant genera only, compared to 332 and 469 for *S. sclerotiorum* and *B. cinerea* respectively (Figure 1A). *S. borealis* host plants include notably *Agropyron, Agrostis, Elymus*, and *Festuca* species that have not been reported as hosts for *S. sclerotiorum* or *B. cinerea* to date. *S. borealis* has an economic impact in countries with cold climates, where it causes snow mold on winter cereals and grasses (Schneider and Seaman, 1987). Its geographic range is restricted to the Arctic Circle, including North of Japan, North America, Scandinavia, and Russia, whereas *B. cinerea* and *S. sclerotiorum* are cosmopolitan fungi found in arctic, temperate and tropical climates (Figure 1B). Consistently, *S. borealis* is a psychrophile, with an optimal growth temperature about 4–10°C, whereas optimal growth temperature is ~23°C for *B. cinerea* and *S. sclerotiorum* (mesophiles) (Wu et al., 2008; Hoshino et al., 2010; Judet-Correia et al., 2010). To successfully thrive in cold environments, psychrophilic pathogens must synthesize enzymes and effectors that perform effectively at low temperatures. Cold-temperature environments present several challenges, in particular reduced reaction rates, increased viscosity, and phase changes of the surrounding medium. A draft genome sequence of *S. borealis* strain F-4128 has recently been released (Mardanov et al., 2014a,b) providing an opportunity to better understand its adaptation to its ecological niche and particularly to cold environment. The total size of the assembled genome of *S. borealis* is 39.3 Mb, with a G+C content of 42%, including 10,171 predicted protein coding sequences (Mardanov et al., 2014a). These characteristics are similar for the genomes of *S. sclerotiorum* 1980 and *B. cinerea* B05.10 with total sizes of 38.3 Mb and 42.3 Mb respectively, G+C content of 41.8 and 43.1% respectively, and 14,503 and 16,448 predicted protein coding genes respectively (Amselem et al., 2011).

Cellular adaptations to low temperatures and the underlying molecular mechanisms are not fully understood but include membrane fluidity, the production of cold-acclimation and antifreeze proteins and maintenance of enzyme-catalyzed reactions and protein-protein interactions involved in essential cellular processes (Feller, 2003; Casanueva et al., 2010). Attempts to correlate protein thermal adaptation with sequence and structure derived features have accumulated with the multiplication of genomic sequencing programs. For instance, analysis of the complete predicted proteome of the psychrophilic bacterium *Colwellia psychrerythraea* supported the view that psychrophilic lifestyle probably involves specific sets of genes in addition to changes in the overall genome content and amino acid composition (Methé et al., 2005). Because microorganisms are at complete thermal equilibrium with their environment, it is indeed conceivable that adaptation to low temperature lead to global alterations of proteomes in psychrophiles. Comparative genomic and metagenomic analyses in prokaryotes demonstrated that the summed frequency of amino acids Ile, Val, Tyr, Trp, Arg, Glu, Leu (IVYWREL) correlates with optimal growth temperature (Zeldovich et al., 2007). In another study on bacteria, Ala, Asp, Ser, and Thr were found preferred in the genome of psychrophiles compared to mesophiles, whereas Glu and Leu are less frequent (Metpally and Reddy, 2009). The analysis of amino acid usage in thermophilic fungi showed that these species indeed have a higher total frequency of IVYWREL amino acids than their mesophilic relatives, but show also significant depletion in Gly and enrichment in Arg and Ala (Van Noort et al., 2013). At the structural level, cold environments seem to release selective pressure for stable proteins, and increase selection for highly active heat-labile enzymes, relying on improved intrinsic disorder to maintain optimal conformation dynamics (Feller, 2003, 2007). Besides these seemingly general principles and given the existence of psychrophiles in lineages across the tree of life, multiple mechanisms contributing to cold adaptation may exist.

For a fungal pathogen such as *S. borealis*, completion of its life cycle requires successful plant colonization, and a subset of secreted virulence factors is likely involved in essential cellular processes. Besides, secreted proteins in both yeasts and mammals were shown to evolve slightly faster than intracellular proteins (Julenius and Pedersen, 2006; Liao et al., 2010), suggesting that the search for signatures of adaptation to *S. borealis* lifestyle may help revealing proteins essential for host and environment colonization in the *Sclerotiniaceae*. In this work, we focused our analysis on adaptations to *S. borealis* environment that lead to alterations in core functions (genes and proteins) conserved in *S. sclerotiorum* and *B. cinerea*. We analyzed a set of 5531 groups of core orthologous proteins for amino acid usage and intrinsic protein disorder patterns specifically associated with *S. borealis* proteins. We highlight a novel putative antifreeze protein and a novel putative lytic polysaccharide monooxygenase identified through our pipeline as candidate proteins involved in colonization of the environment. Our findings suggest that similar protein signatures associate with *S. borealis* lifestyle and with secretion in the *Sclerotiniaceae*. These signatures may be useful for identifying proteins of interest as targets for the management of plant diseases and for the bio-conversion of plant biomass.

## Results

### A pipeline to reveal *S. borealis* protein sequence signatures in multiple ortholog alignments

**Figure 2:**
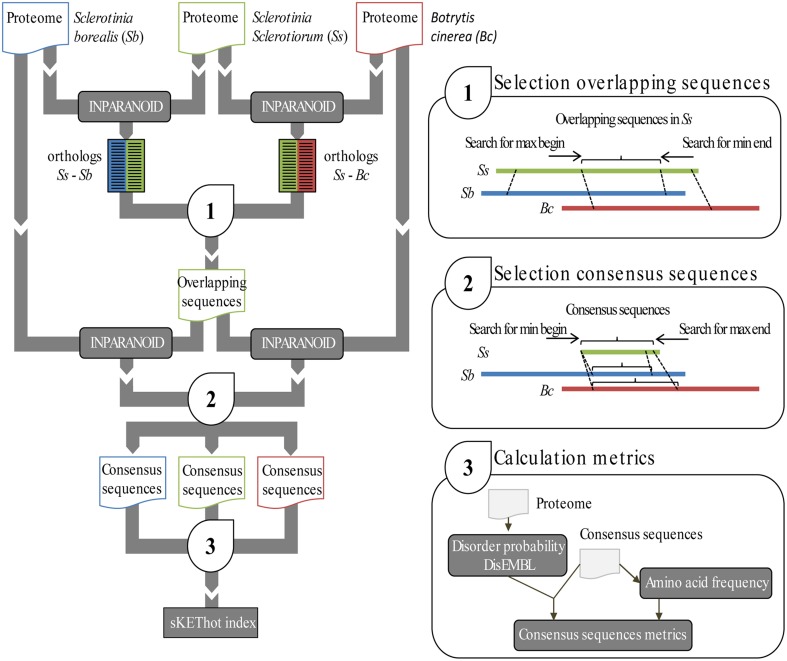
**Bioinformatics pipeline for the identification of *S. borealis* protein sequence signatures in multiple ortholog alignments**. Our pipeline uses complete predicted proteomes of *S. borealis, S. sclerotiorum*, and *B. cinerea* as inputs. It identifies orthologous protein pairs in *S. borealis* and *S. sclerotiorum*; and in *S. borealis* and *B. cinerea* using Inparanoid. Using *S. sclerotiorum* proteins as a reference, it identifies non-redundant core ortholog groups (COG) and overlapping regions (1). A second Inparanoid run is then used to define the longest aligned region in all three genomes (“consensus”) for each COG (2). Next, protein sequence metrics (disorder probability and amino acid frequencies) are calculated for consensus regions of all proteins (3). Finally, Wilcoxon sum rank tests are performed to identify metrics significantly different in *S. borealis* proteins.

Several studies reported specific amino acid usage patterns and intrinsic disorder frequency in proteins from psychrophilic bacteria as compared to related mesophilic bacteria (Methé et al., 2005; Metpally and Reddy, 2009). To test whether *S. borealis* proteins have a distinctive pattern of amino acid usage and disorder compared to *S. sclerotiorum* and *B. cinerea* proteins, we designed a bioinformatics pipeline to process complete proteomes deduced from the whole genome sequences of these three fungal pathogens (Figure 2) (Amselem et al., 2011; Mardanov et al., 2014a). To exclude patterns that may be due to factors unrelated to adaptation in *S. borealis* proteins, we focused our analysis on core groups of orthologous proteins with one member from each species. A total of 6717 core orthologous groups (COGs) were identified using two pairwise InParanoid proteome comparisons (Ostlund et al., 2010) as explained in material and methods section and presented in Figure 2, covering between ~42% (*B. cinerea*) to ~66% (*S. borealis*) of complete predicted proteomes. We used multiple alignments of the three proteins in each COG to select *S. sclerotiorum* protein regions conserved in *S. borealis* and *B. cinerea*. To retrieve core protein regions conserved in all three members of COGs, we ran another round of InParanoid pairwise comparisons using conserved regions of *S. sclerotiorum* proteins as input. Short alignments can artificially cause strong variations in amino acid proportions. To reduce this confounding effect, we excluded alignments producing a consensus sequence shorter than 200 amino acids. We obtained a total of 5531 COG alignments matching these criteria that were processed for amino acid frequency and intrinsic protein disorder analysis.

### *S. borealis* proteins show specific intrinsic disorder and amino acid usage patterns compared to their *sclerotiniaceae* orthologs

**Figure 3:**
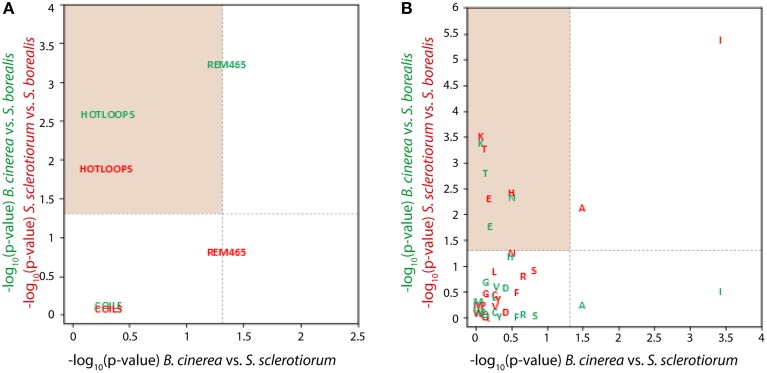
**Adaptation to *S. borealis* lifestyle associates with specific amino acid usage and protein disorder patterns**. Distribution of the p-values of Wilcoxon sum rank tests performed to identify intrinsic disorder probabilities **(A)** and amino acid frequencies **(B)** that are significantly different in *S. borealis* core orthologs. For each amino acid frequency and intrinsic disorder probability, three pairwise tests were performed to compare (i) values in *B. cinerea* and *S. sclerotiorum* orthologs (*p*-values shown along the X-axis), (ii) values in *S. borealis* and *B. cinerea* orthologs (*p*-values shown along the Y-axis in green), and (iii) values in *S. borealis* and *S. sclerotiorum* orthologs (*p*-values shown along the Y-axis in red). Amino acid frequencies and intrinsic disorder probabilities that fell in the shaded areas were considered significantly different between *S. borealis* and the other fungi (*p* < 0.05) but not between *S. sclerotiorum* and *B. cinerea* (*p*>0.05). These properties were considered as associated with *S. borealis* lifestyle.

To document intrinsic protein disorder and amino acid usage in *Sclerotiniaceae* COGs, we calculated frequencies of each of the 20 amino acids in the aligned protein regions as well as their disorder frequencies. Determination of the disorder frequencies were obtained by assigning to each amino acid of the aligned protein regions their disorder probability obtain by submitting the full length protein to disEMBL analyses (Linding et al., 2003). The disEMBL output contained three measures of intrinsic protein disorder designated as “Coils,” “Hot loops,” and “Remark465” corresponding to their probability to be involved in disorder region. To test whether any of these 20 amino acid frequencies plus 3 disorder metrics frequencies showed a significantly different distribution in *S. borealis* COG aligned regions compared to *S. sclerotiorum* and *B. cinerea*, we performed pairwise Wilcoxon sum rank tests to compare distributions of each of the 23 properties in *S. borealis* and *S. sclerotiorum*, in *S. borealis* and *B. cinerea*, and in *S. sclerotiorum* and *B. cinerea* (Table S1). We considered that a protein property was significantly associated with *S. borealis* COG aligned regions when Wilcoxon sum rank tests were significant (*p* < 0.05) for *S. borealis*—*S. sclerotiorum* and *S. borealis*—*B. cinerea* comparisons but not (*p*>0.05) for *S. sclerotiorum*—*B. cinerea* comparison. The “hot loops” frequencies measure of intrinsic protein disorder was found significantly associated with *S. borealis* COG aligned regions, whereas “Coils” and “Remark465” were not (Figure 3A). “Hot loops,” corresponding to protein regions predicted not to adopt helix or strand secondary structure and having a high degree of flexibility, were found significantly depleted in *S. borealis* COG aligned regions. *S. borealis* proteins had a median hot loop frequency of 3.43% in COG aligned regions, vs. 3.67% in *S. sclerotiorum* and 3.71% in *B. cinerea* proteins. Regarding frequency of amino acids, three were found significantly associated with *S. borealis* aligned COG regions. Thr frequency was significantly enriched, representing 6.00% of amino acids in *S. borealis* instead of 5.93% in *S. sclerotiorum* and 5.91% in *B. cinerea* proteins. Lys and Glu were significantly depleted in *S. borealis*. Lysine represented 5.26% of amino acids in *S. borealis* instead of 5.41% in *S. sclerotiorum* and *B. cinerea* proteins; Glu represented 6.43% of amino acids in *S. borealis* instead of 6.54% in *S. sclerotiorum* and 6.57% in *B. cinerea* proteins (Figure 3B). These findings are consistent with the view that cold adaptation includes the directional adaptation of pre-existing protein functions (intrinsic protein structure and amino acid composition) in addition to specific sets of genes conferring a psychrophilic lifestyle, such as previously reported in bacteria (Methé et al., 2005).

### The distribution of sTEKhot index is biased in *S. borealis* orthologous proteins and complete predicted proteome

**Figure 4:**
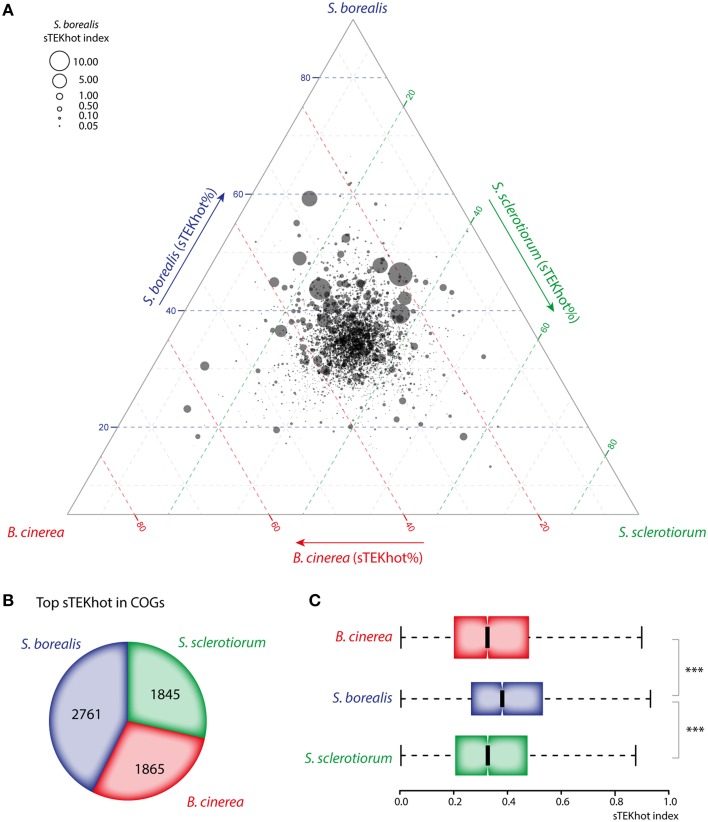
**The sTEKhot index discriminates *S. borealis* proteins in core ortholog groups and whole predicted proteomes**. **(A)** Overall distribution of sTEKhot values from the three fungal species within COGs. Each bubble represents a COG positioned according to the contribution of each ortholog (sTEKhot%) to the total sTEKhot of the COG. Therefore, orthologs that have similar sTEKhot values in all three species appear at the center of the plot, while COGs appear near the corner of the species harboring the ortholog with the highest sTEKhot otherwise. The size of bubbles is proportional to the sTEKhot value of *S. borealis* orthologs. Data points are frequent above the 40% line for *S. borealis* sTEKhot, and less so for *S. sclerotiorum* and *B. cinerea* sTEKhot indicating frequent higher sTEKhot values in *S. borealis* orthologs. **(B)** Species distribution of orthologs having the highest sTEKhot value in COGs. **(C)** Distribution of the sTEKhot index in the whole predicted proteome of the three fungi.

Several studies reported biases in amino acid usage in the proteome of extremophiles and proposed indices able to discriminate proteins from extremophilic and related mesophilic organisms (Suhre and Claverie, 2003; Zeldovich et al., 2007; Wang and Lercher, 2010). To analyze the degree to which intrinsic protein disorder and amino acid usage of individual proteins matches with specific patterns identified in *S. borealis* predicted proteome, we designed the *S. borealis* T (Thr), E (Glu), K (Lys), hot (hot loops) index as follows:

(1) $$\begin{array}{r} sTEKhot=\frac{T}{E+K+hot} \end{array}$$

where “T,” “E,” and “K” are the normalized frequencies of Thr, Glu and Lys respectively in a given protein sequence, and “hot” is the normalized frequency of hot loops in this sequence. We computed the sTEKhot index for each protein in the predicted proteomes of S. *borealis, S. sclerotiorum*, and *B. cinerea*. First, we compared the distribution of sTEKhot values in COGs by plotting all values in a ternary plot (Figure 4A). This revealed that sTEKhot values are distributed along an axis pointing toward *S. borealis* angle, clearly showing that sTEKhot values of *S. borealis* orthologs are major contributors to the structure of the dataset. There was 692 COGs in which *S. borealis* sTEKhot value accounted for >40% of the total sTEKhot for the COG, but only 388 and 345 in which *S. sclerotiorum* and *B. cinerea* sTEKhot values respectively accounted for >40% of the total sKTEHhot for the COG (Figure 4A). Consistently, *S. borealis* has the highest sTEKhot value in 42.7% of COGS (2761), whereas *S. sclerotiorum* and *B. cinerea* have the highest sTEKhot value in 28.3% (1845) and 28.8% (1865) of the COGs respectively (Figure 4B).

At the whole proteome level, sTEKhot median was 0.366 in *S. borealis*, but only 0.314 in *S. sclerotiorum* and 0.313 in *B. cinerea* (Figure 4C, Table S2). The overall sTEKhot distributions were significantly different when comparing *S. borealis* to the two other species (*p* < 5.1.e^−104^) but not when comparing *S. sclerotiorum* to *B. cinerea* (*p* = 0.84). However, a subset of *S. sclerotiorum* and *B. cinerea* proteins appeared to have high sTEKhot values. Indeed, as mentioned previously, *S. sclerotiorum* and *B. cinerea* each account for the highest sTEKhot in ~30% of the COGs. Furthermore, the proportion of proteins with a sTEKhot > 1 was 6.2% in *S. borealis*, 4.6% in *S. sclerotiorum* and 5.0% in *B. cinerea*. This suggests that the general pattern of intrinsic protein disorder and amino acid usage observed in *S. borealis* protein may be shared by a subset of *S. sclerotiorum* and *B. cinerea* predicted proteome.

To verify that the sTEKhot index was an optimized combination of intrinsic protein disorder and amino acid usage measures to discriminate the proteome of *S. borealis* from that of *S. sclerotiorum* and *B. cinerea*, we randomly shuffled the 23 measures for intrinsic protein disorder and amino acid usage in equation (1) and calculated the proteome median value for shuffled indices in *S. borealis, S. sclerotiorum*, and *B. cinerea* (Table S3). In 300 shuffling iterations, the *p*-value for difference between the distribution of shuffled index in *S. borealis* and *S. sclerotiorum* or *B. cinerea* was < 5.1.e^−104^ (highest observed *p*-value) in only 6 instances. The median shuffled index value for *S. borealis* proteome was higher than the observed sTEKhot median in only 2 instances over 300 (0.6%). Wilcoxon ranking tests comparing random medians distribution to real sTEKhot median showed *p* < 4.72e^−33^ in the three species. The result of these simulations indicate that sTEKhot clearly departs from random in describing specific intrinsic protein disorder and amino acid usage patterns in *S. borealis* proteins.

### Secreted enzymes are enriched among *S. borealis* proteins with high sTEKhot

**Figure 5:**
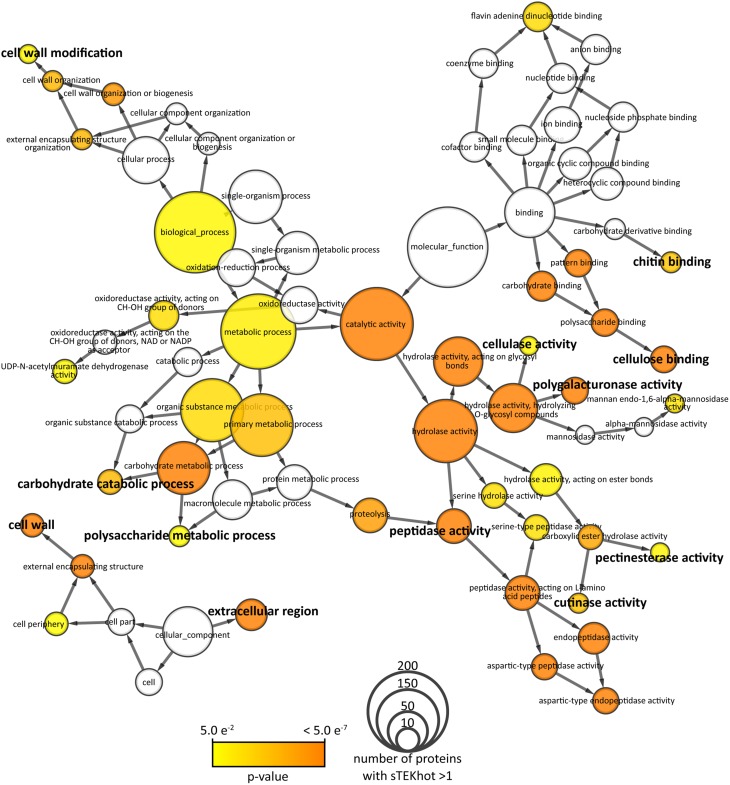
**Network representation of gene ontologies (GOs) of proteins with sTEKhot >1 in *S. borealis* proteome**. Nodes correspond to GOs are sized according to the number of proteins with sTEKhot >1. They are colored from yellow to orange according to the *p*-value of a hypergeometric test for enrichment in proteins with sTEKhot >1 compared to whole proteomes. White nodes are GOs not significantly enriched among proteins with sTEKhot > 1 (*p*>0.05). GOs labeled in bold font correspond to functions possibly associated with host interaction.

To identify protein functions important for adaptation to *S. borealis* environment, we analyzed annotations of proteins with a sTEKhot value higher than 1 in *S. borealis* proteome. Overall, 4794 (47%) *S. borealis* proteins had no Gene Ontology (GO) annotation assigned. There were 635 proteins with sTEKhot > 1, among which 349 (55%) had no GO annotation. We looked for GO term enrichment in the 635 *S. borealis* with sTEKhot > 1 compared to all annotated proteins. Forty two GO terms appeared significantly enriched (*p* < 0.05) among proteins with sTEKhot > 1, including 16 leaves (GO with no child term) of the GO network (Figure 5). GO terms for “cellular component” enriched in proteins with sTEKhot > 1 included extracellular and cell wall compartments. Consistently, enriched “biological processes” and “molecular functions” related to secreted enzymes involved in cell wall modification (glycosyl hydrolases and carboxylic ester hydrolases, among which are pectinesterases and cutinases) and binding to cellulose. Cellulose is a major component of plant cell walls that fungal pathogens are able to detect and bind. Also plants aerial parts are protected by a cuticle composed by cutin. Fungal pathogens are able to hydrolyze cutin through cutinases, thus facilitating host colonization. In addition, proteins involved in carbohydrate metabolism were enriched among proteins with sTEKhot > 1. These functions are associated with colonization of the environment, especially plant-associated environment. Similar enrichments where observed when looking at GO annotations for *S. sclerotiorum* and *B. cinerea* proteins harboring a sTEKhot > 1 (Figures S1, S2). In addition, copper ion binding GO was found to be enriched in *S. sclerotiorum* and *B. cinerea*.

### Secreted proteins have higher sTEKhot than non-secreted proteins in the three *Sclerotiniaceae* species

**Figure 6:**
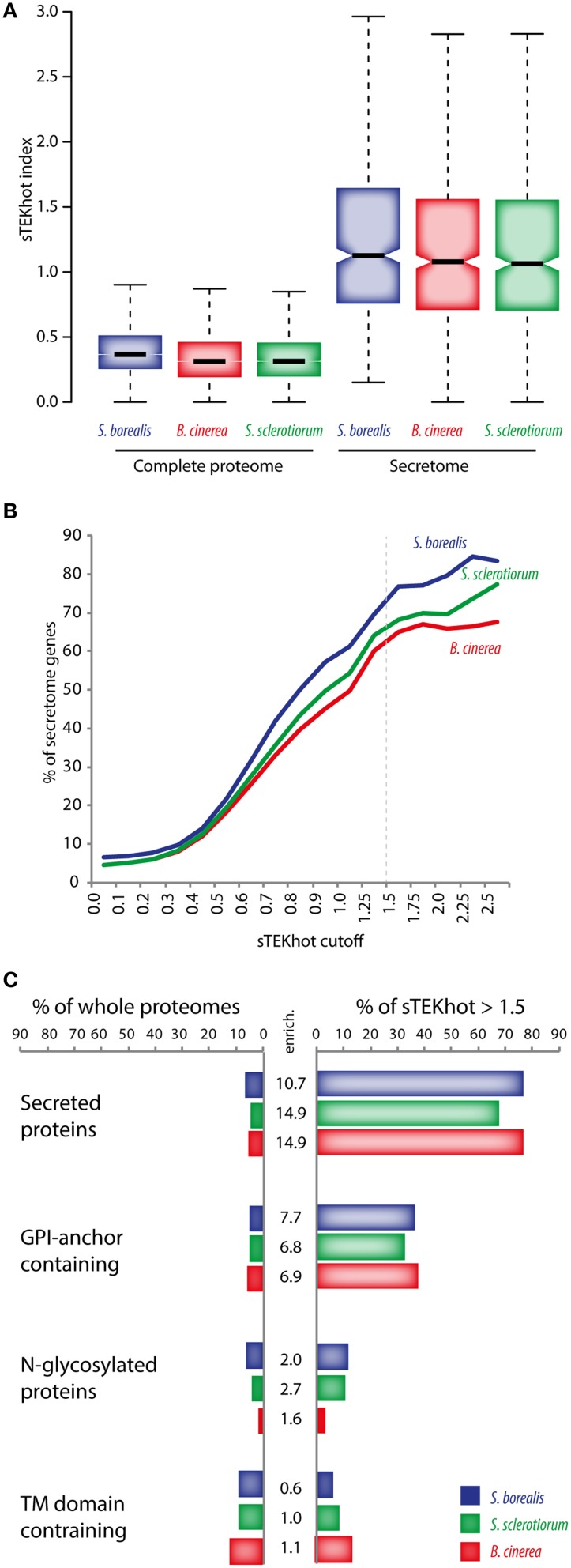
**Predicted secreted proteins have high sTEKhot values. (A)** Distribution of sTEKhot values in the proteome and the secretome of *S. borealis, S. sclerotiorum* and *B. cinerea*. **(B)** Proportion of predicted secreted proteins according to sTEKhot cutoff values. In complete proteomes (sTEKhot ≥ 0), the proportion of secreted proteins is ~5% in all three fungal proteomes, whereas among proteins with sTEKhot ≥ 1.5 (dotted line) it reaches an average ~70%. **(C)** Proportion of whole proteomes and proteins with sTEKhot > 1.5 that are secreted, contain GPI-anchors, are N-glycosylated or contain transmembrane (TM) domains. Enrich., enrichment fold among sTEKhot > 1.5 as compared to whole proteomes.

The enrichment of extracellular proteins among proteins with sTEKhot > 1 prompted us to compare the distribution of sTEKhot for secreted and non-secreted protein in the *Sclerotiniaceae*. We considered as predicted secreted proteins those identified as secreted with SignalP 4.0 no-TM network and as extracellular by WoLF PSORT. This produced lists of 667, 661, and 748 predicted secreted proteins (secretome) for *S. borealis, S. sclerotiorum*, and *B. cinerea* respectively. In all three fungal species, secreted proteins had significantly higher sTEKhot values than non-secreted proteins, with median sTEKhot values for secreted proteins of 1.13 in *S. borealis*, 1.06 in *S. sclerotiorum* and 1.08 in *B. cinerea* (Figure 6A). The distribution of sTEKhot in secreted proteins was found significantly higher than its distribution in non-secreted proteins with *p*-value of 8.8e^−239^ in *S. borealis*, 9.1e^−265^ in *S. sclerotiorum* and 4.1e^−275^ in *B. cinerea* respectively. To evaluate the likelihood of obtaining such distributions with other intrinsic protein disorder and amino acid usage parameters, we randomly shuffled the 23 measures for intrinsic protein disorder and amino acid usage in Equation (1), and calculated shuffled indices for each protein in the predicted secretome in the three species. In 300 rounds of shuffling, the median secretome index was found higher than the observed median secretome sTEKhot in 3, 1 and 1 instance for *S. borealis, S. sclerotiorum* and *B. cinerea* respectively (Table S3).

Remarkably, although secreted proteins account for 6.5% of total proteome in *S. borealis*, 4.5% in *S. sclerotiorum* and 4.5% in *B. cinerea*, the proportion of secreted proteins among those with sTEKhot > 1.5 raised to 76.9% (206 out of 268) in *S. borealis*, 68.2% (182 out of 267) in *S. sclerotiorum* and 65.0% (206 out of 317) in *B. cinerea*, representing ~13.6 fold enrichment in secreted proteins (Figure 6B). These results suggest that intrinsic protein disorder and amino acid usage patterns associated with *S. borealis* lifestyle and secretion are largely overlapping in the *Sclerotiniaceae*.

To independently validate this observation, we compared the distribution of all amino acid frequencies and the distribution of the three intrinsic protein disorder measures used previously in secreted and non-secreted proteins from the three fungal species. We considered that a protein property is associated with secretion when the null hypothesis of the Wilcoxon sum-rank test (distribution of property no different between secreted and non-secreted proteins) could be rejected with *p* < 0.05 for all three fungal species. Among the 23 measures for protein disorder and amino acid usage, 21 could be significantly associated with fungal secretomes, supporting the view that function outside the cell imposes specific constraints on amino acids usage in secreted proteins, such as evolution toward reduced synthetic cost of proteins (Smith and Chapman, 2010). Similar to patterns associated with *S. borealis* lifestyle, we found that enrichment in Thr, depletion in Glu and reduced frequency of hot loops disorder are among the properties most significantly associated with secretion (*p*-values ranging from 7.62e^−3^ to 2.67e^−194^) (Table S4).

We considered several hypotheses to explain the observed common signatures for *S. borealis* lifestyle and secretion. First, we envisaged that prevalence of secreted proteins in COGs may have biased signatures of *S. borealis* lifestyle toward properties associated with secretion. However, ratios of secreted proteins in COG sets were similar to those observed for total proteomes (7% in *S. borealis*, 6.7% in *S. sclerotiorum* and 6.4% in *B. cinerea* proteins from the set of 5531 COGs). Furthermore, we excluded COGs that comprised secreted proteins and tested whether amino acid usage patterns associated with *S. borealis* proteins as previously. Amino acids enriched in *S. borealis* proteins included Thr and amino acids depleted in *S. borealis* included Glu and Lys (*p* < 0.05), similar to what we found in our initial analysis taking all COGs into account. In addition, we also found His enriched in *S. borealis* sequences and Asn depleted (*p* < 0.05). We conclude that the detection of a bias in the usage of these amino acids in *S. borealis* proteins was not due to the abundance of secreted proteins in COGs (Table S5). Second, we hypothesized that intrinsic protein disorder and amino acid usage in secreted proteins might be due to signal peptide regions. To test this, we analyzed protein properties associated with mature secreted proteins (signal peptide region removed). We found that mature secreted proteins had significantly higher sTEKhot than the rest of the proteome (*p* < 2.4.e^−232^), similar to what we found with full length secreted proteins (Figure S3). Therefore high sTEKhot in secretomes is not due to signal peptide sequence. Third, we considered that high sTEKhot in secretomes could arise if secretomes were be less divergent than the rest of the proteomes, leading to *S. borealis* signature being more conserved in secreted proteins of *S. sclerotiorum* and *B. cinerea*. To test this, we analyzed the distribution of similarity between *S. borealis* proteins and their closest homologs in *S. sclerotiorum* and *B. cinerea*. Whereas the average BLASTP score was 630.9 for *S. borealis* non-secreted proteins aligned with their closest homolog in *S. sclerotiorum*, this average score was 521.6 for *S. borealis* secreted proteins (Figure S4). This indicates that globally, *S. borealis* secretome is more divergent from *S. sclerotiorum* proteome than *S. borealis* non-secreted proteins. A similar tendency was observed when comparing *S. borealis* and *B. cinerea* proteomes. The high sTEKhot average observed in *Sclerotiniaceae* secretomes is therefore not due to higher similarity in secretomes compared to non-secreted proteins.

To test whether proteins with high sTEKhot could be enriched in other types of motifs, we predicted glycosylphosphatidylinositol (GPI) anchors, transmembrane (TM) domains and N-glycosylation sites in the proteome of *S. borealis, S. sclerotiorum* and *B. cinerea*. We found an average of 5.0% of proteins with GPI-anchors, 9.9% proteins with TM domains and 3.8% of proteins with >10 predicted N-glycosylation sites in the *Sclerotiniaceae* species (Table S6, Figure 6C). As compared to whole proteomes, the list of proteins with sTEKhot >1.5 showed an average 7.1-fold enrichment in proteins with GPI-anchors, 2.1-fold enrichment in proteins with >10 predicted N-glycosylation sites and no enrichment in proteins with TM domains (Figure 6C). Secreted proteins showed the strongest enrichment among proteins with sTEKhot >1.5. Overall these analyses suggest that a significant overlap exists between the constraints imposed on protein sequence by adaptation to *S. borealis* lifestyle and to secretion in the *Sclerotiniaceae*.

### *S. sclerotiorum* genes encoding proteins with high sTEKhot are enriched in genes induced *in planta*

**Figure 7:**
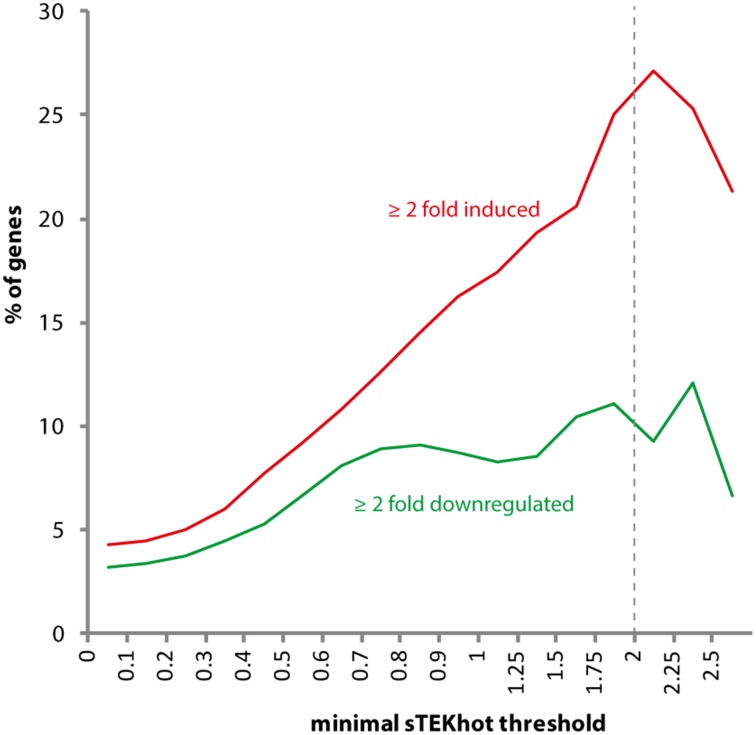
**Proportion of *S. sclerotiorum* proteins encoded by genes differentially expressed *in planta* according to sTEKhot cutoff values**. In *S. sclerotiorum* complete genome (sTEKhot ≥ 0), the proportion of genes induced ≥2-fold *in planta* is ~4.31%, whereas it reaches ~27.1%.among proteins with sTEKhot ≥ 2 (dotted line).

To further support the association between high sTEKhot index and colonization of the environment, and particularly host plants, we analyzed the distribution of sTEKhot values in *S. sclerotiorum* genes differentially regulated *in planta*. For this, we took advantage of *S. sclerotiorum* microarray gene expression data generated by Amselem *et al*. from infected sunflower cotyledons (Amselem et al., 2011). In this dataset, out of 14 503 predicted protein coding genes, 615 were induced at least two-fold during infection of sunflower (4.31%) and 458 genes down-regulated at least two-fold (3.21%). The proportion of genes induced *in planta* reached 27.1% of *S. sclerotiorum* genes encoding proteins with sTEKhot ≥ 2, representing ~6.3-fold enrichment (Figure 7). The proportion of genes down-regulated *in planta* reached 12.1% of *S. sclerotiorum* genes encoding proteins with sTEKhot ≥ 2, representing ~3.8-fold enrichment. *S. sclerotiorum* proteins with sTEKhot > 1 include six proteins with CFEM domain, a Cys-rich domain with proposed role in fungal pathogenesis, two proteins with a cerato-platanin domain, one of which being the ortholog of *B. cinerea* pathogen associated molecular pattern BcSpl1 (Frías et al., 2011), 27 proteins with a pectin lyase fold found in *Aspergillus* virulence factors (Mayans et al., 1997), and 29 out of 78 effector candidates proposed by Guyon et al. (2014). These findings are consistent with important role in the colonization of the host plant for some proteins with high sTEKhot values.

### High sTEKhot index and secretion signal reveal candidate proteins associated with colonization of the environment

**Figure 8:**
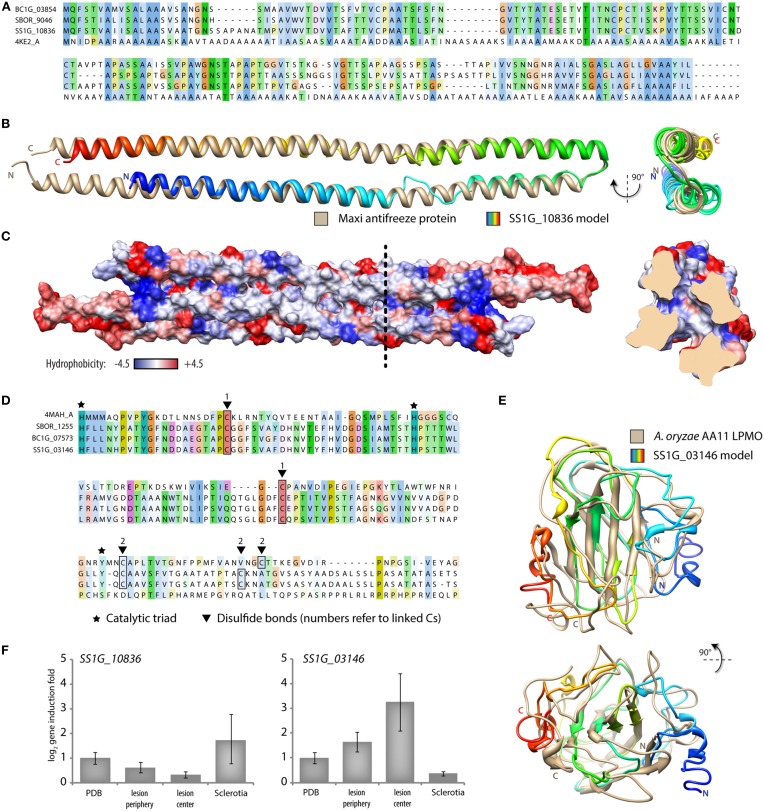
**Candidate proteins associated with colonization of the environment identified based on high sTEKhot values**. **(A)** Multiple protein sequence alignment of *B. cinerea* BC1G_03854 (sTEKhot = 4.29), *S. borealis* SBOR_9046 (sTEKhot = 10.01), *S. sclerotiorum* SS1G_10836 (sTEKhot = 7.34) and the hyperactive Type I antifreeze protein “Maxi” from *Pseudopleuronectes americanus* (4KE2_A). **(B)** Superimposition of Maxi antifreeze protein structure (tan) and SS1G_10836 model structure (rainbow). **(C)** Surface hydrophobicity of SS1G_10836 model dimer. Dotted line corresponds to the position of the section shown on the right, illustrating the characteristic hydrophilic inner core of the dimer. **(D)** Multiple protein sequence alignment of *B. cinerea* BC1G_07573 (sTEKhot = 7.07), *S. borealis* SBOR_1255 (sTEKhot = 3.79), *S. sclerotiorum* SS1G_03146 (sTEKhot = 1.58) and the AA11 Lytic Polysaccharide Monooxygenase from *Aspergillus oryzae* (4MAH_A). **(E)** Superimposition of *A. oryzae* AA11 structure (tan) and SS1G_03146 model structure (rainbow). **(F)** *SS1G_10836* and *SS1G_03146* gene expression *in vitro* (PDB, Potato Dextrose Broth), during colonization of *Arabidopsis thaliana* (lesion periphery and lesion center) and in sclerotia. Error bars show standard error of the mean from two independent biological replicates.

To illustrate the value of the sTEKhot index for the exploration of the proteome of fungi from the *Sclerotiniaceae*, we analyzed in detail the sequence of two proteins with high sTEKhot but with no assigned function. Over the three proteomes analyzed, *S. borealis* SBOR_9046 had the highest sTEKhot (10.01). In *S. sclerotiorum*, its ortholog is SS1G_10836 which ranked as the 5th highest sTEKhot in *S. sclerotiorum* (7.34). In *B. cinerea*, its ortholog is BC1G_03854 which ranked as the 23rd highest sTEKhot in *B. cinerea* (4.29). No interproscan domain or GO terms were associated with these proteins of 171 amino acids (except SS1G_10836 which is 173 amino acids long). To get insights into their putative function, we performed protein structure modeling and fold recognition using the I-TASSER server (Zhang, 2008). The closest structural analog was the antifreeze protein Maxi from winter flounder (*Pseudopleuronectes americanus*) (Sun et al., 2014). Although sequence similarity with Maxi was limited (from 15.2% identity for SBOR_9046 to 16.2% identity for SS1G_10836), superimposition of SS1G_10836 predicted structure with Maxi structure showed a Root Mean Square Deviation < 2.3Å and a TM-score of 0.875, indicating structural similarity deviating significantly from random (Figures 8A,B). Analysis of SBOR_9046, SS1G_10836 and BC1G_03854 sequence by TargetFreeze (He et al., 2015) supported the prediction as antifreeze proteins. The *Sclerotiniaceae* proteins contain four Cys residues located in the kink of predicted structures that may stabilize folding like, although these residues were not predicted to form disulfide bonds by Disulfind (Ceroni et al., 2006). Antifreeze proteins have been reporting that rely on disulfide bonds for folding (Basu et al., 2015) whereas others do not (Kondo et al., 2012; Sun et al., 2014). Like other known fungal antifreeze proteins (Kondo et al., 2012), but unlike Maxi, SBOR_9046 and its orthologs are predicted to be secreted. A unique feature of Maxi among antifreeze proteins is the presence of ice-binding residues buried within the four-helix bundle instead of exposed on their surface (Sun et al., 2014). A prediction of SS1G_10836 dimer structure supports the existence of rather hydrophilic pockets buried within the four-helix bundle, suggesting that the mechanism of ice binding of Maxi could be conserved in SS1G_10836 and its orthologs (Figure 8C). To get insights into SS1G_10836 function, we analyzed the expression of the corresponding gene in mycelium grown in Potato Dextrose Broth (PDB), during the colonization of *Arabidopsis* plants and in sclerotia by quantitative RT-PCR. This revealed a 3.3-fold induction (log_2_ = 1.7) specific to sclerotia (Figure 8F). Since sclerotia overwinter in the soil, putative antifreeze proteins may contribute to survival of these structures both in arctic and temperate climates.

The COG including SS1G_03146, BC1G_07573, and SBOR_1255 is remarkable for including three proteins with high (>1) but with very variable sTEKhot, ranging from 1.58 (SS1G_03146) to 7.07 (BC1G_07573). No interproscan domain or GO terms were associated with these proteins of 223 amino acids in average, but all three were predicted to include a N-terminal signal peptide for secretion. To get insights into their putative function, we performed protein structure modeling and fold recognition using the I-TASSER server (Zhang, 2008). The closest structural analog was *Aspergillus oryzae* AA11 (AoAA11) Lytic Polysaccharide Monooxygenase (LPMO) (Hemsworth et al., 2014). Sequence similarity with AoAA11 was limited (from 9.6% identity for SBOR_1255 to 10.9% identity for SS1G_03146), superimposition of SS1G_03146 predicted structure with AoAA11 structure showed a Root Mean Square Deviation < 3.1Å and a TM-score of 0.677, indicating structural similarity deviating significantly from random (Figures 8D,E). Similar to the *Sclerotiniaceae* proteins, full length AoAA11 (accession number XM_001822611) harbors a N-terminal signal peptide. AoAA11, SBOR_1255, and BC1G_07573 feature two conserved predicted disulfide bonds, SS1G_03146 is predicted to contain only one (Figure 8D). The catalytic triad of AoAA11 appears nicely conserved in the *Sclerotiniaceae* proteins, with the exception of the catalytic Tyr replaced by a Ser in SS1G_03146 (Figure 8D). LPMOs are enzymes oxidizing recalcitrant polysaccharides such as cellulose, starch and chitin. They present excellent potential for use in biomass conversion and the production of biofuels. *Aspergillus oryzae* AA11 represents a new class of LPMOs that include a putative chitin-binding domain (Hemsworth et al., 2014). We analyzed the expression of the *SS1G_03146* gene in mycelium grown in PDB, during the colonization of *Arabidopsis* plants and in sclerotia by quantitative RT-PCR. This revealed up to 9.5-fold induction (log_2_ = 3.25) during plant infection (Figure 8F). This suggests that SS1G_03146 may be involved in colonization of the plant, but functional analysis will be required to determine its actual role.

Based on these predicted functions, we propose that SS1G_10836 and SS1G_03146 have important functions in the colonization of the environment, the identification of which was facilitated by the implementation of the sTEKhot index. Functional studies will be required to test predicted functions of these proteins. Furthermore, these two proteins have predicted properties that may be exploited for biotechnology purposes.

## Discussion

Understanding how fungal plant pathogens colonize their environment, including their host plants, is critical for food security and the sustainable management of ecosystems (Roux et al., 2014). In particular *B. cinerea* and *S. sclerotiorum* are threatening hundreds of plant species and important crop species in the majority of regions of the globe. Fungi also represent a remarkable reservoir of enzymes with very diverse catalytic abilities that are employed in industrial processes. We have conducted a comparative analysis of the proteome and secretome of fungal species from the *Sclerotiniaceae* revealing common principles of sequence optimization for secreted proteins.

In the present study we designed a bioinformatics pipeline aiming at identifying species-specific patterns of amino acid usage and intrinsic protein disorder in the proteome of closely related species. We applied this pipeline to agriculturally important fungal pathogens from *Sclerotiniaceae* family to reveal specific signatures associated with *S. borealis* lifestyle. Compared to *S. sclerotiorum* and *B. cinerea* orthologs, we observed in *S. borealis* proteins a significant increase in Thr usage and a significant decrease in Glu and Lys usage. To minimize the impact of phylogenetic distance on the definition of *S. borealis* sequence signature, we have restricted our analysis to species from the *Sclerotiniaceae* family and we discarded any sequence signature differing significantly between *S. sclerotiorum* and *B. cinerea.* It is also worth noting that *S. borealis, S. sclerotiorum* and *B. cinerea* have a very similar G+C content, so that G+C bias is not expected to have an impact on the differential usage of amino acids. Specific trends in amino acid composition have been reported to associate with protein stability at extreme temperatures. Given the diversity of ecological groups including psychrophiles, it has been challenging to identify universal trends in amino acids usage associated with cold adaptation (Casanueva et al., 2010). Enrichment in Thr has been reported in solvent-accessible areas of proteins from two cold-adapted Archaea (Goodchild et al., 2004) and in proteins from several psychrophilic bacteria (Metpally and Reddy, 2009). This was proposed to reduce surface charge while minimizing risk of aggregation (Goodchild et al., 2004). Frequent substitutions of Glutamate were observed in exposed sites of selected psychrophilic enzymes (Gianese et al., 2001) and more generally in the proteome of the psychrophilic Archea *Halorubrum lacusprofundi* (Dassarma et al., 2013). Glu is also part of a set of amino acids shown to correlate significantly with optimal growth temperature of prokaryotes (Zeldovich et al., 2007). Specific signatures of amino acid usage we found in *S. borealis* are therefore consistent with some previous observations made for psychrophilic proteins. Nevertheless, our approach does not allow dissociating psychrophily and other specific life traits of *S. borealis* (specific host range, geographic habitat) as drivers of the observed protein signatures. We observed a reduction in the frequency of intrinsic disorder in hot loops in *S. borealis* proteins. By contrast, cold adapted enzymes were often reported to harbor low conformational stability to maintain high reaction rates at low temperature (Feller, 2007; Casanueva et al., 2010) and intrinsically disordered proteins were shown to be more resistant to cold than globular proteins (Tantos et al., 2009). A global study of intrinsic protein disorder in 332 prokaryotes showed however that psychrophilic bacteria have a lower level of intrinsic disorder than mesophiles, although this was proposed to be due to the loss of cellular functions relying on intrinsically disordered proteins (Burra et al., 2010). This analysis also supports the view that adaptations to *S. borealis* lifestyle include directional changes in the sequence of conserved proteins, in addition to possible gene gains and losses that have not been analyzed in this work.

Enrichment analyses revealed that signatures associated with *S. borealis* lifestyle are frequent in plant cell wall degrading enzymes, carbohydrate binding domain containing proteins and ion binding proteins. More generally, secreted proteins showed high sTEKhot values in *S. borealis, S. sclerotiorum* and *B. cinerea*. The proportion of predicted secreted proteins reaches over 75% of *S. borealis* proteins with sTEKhot > 1.5 and the proportion of proteins encoded by *in-planta* induced genes reaches over 27% of *S. sclerotiorum* proteins with sTEKhot > 2, suggesting that sTEKhot may be a useful criterion to identify proteins associated with environmental adaptation or potential virulence factors. More specifically, there were 117 proteins predicted to be secreted and harboring a sTEKhot > 1.5 with no annotation in *S. sclerotiorum* that could include uncharacterized virulence factors. Although some classes of protein effectors from bacteria and oomycete pathogens can be identified relatively easily thanks to conserved N-terminal sequence signals, this strategy has proven limited for fungal pathogens. Alternative bioinformatics approaches have been developed exploiting known effector properties for searching effector candidates in the secretome of fungal pathogens (Saunders et al., 2012; Guyon et al., 2014). Typical effector properties include the presence of a N-terminal secretion signal, small protein size, high Cys content, the absence of characterized protein domains, high rate of non-synonymous over synonymous substitutions (Hacquard et al., 2012; Saunders et al., 2012; Persoons et al., 2014; Sperschneider et al., 2014). However, validated virulence factors do not all comply with these properties, such as *Verticillium dahlia* isochorismatase VdIsc1 harboring an isochorismatase domain but no conventional secretion signal (Liu et al., 2014) or *Melampsora lini* AvrM that lacks any Cys (Catanzariti et al., 2006).

Amino acid composition is a feature used to predict candidate bacterial effectors. Positive charge, richness in alkaline (H, R, K) amino acids and Glu in the 30 C-terminal amino acids is for instance a property often found in type IV secreted effectors (Meyer et al., 2013; Zou et al., 2013; Wang et al., 2014). In *Pseudomonas syringae*, amino acid biases and patterns at the N-terminus were used to identify type III effector candidates. Enrichment in Thr and depletion in Leu is a characteristic of bacterial type III proteins secreted into animal and plant cells, although high sequence variability and high tolerance of mutations make these properties difficult to generalize (Arnold et al., 2009; McDermott et al., 2011; Schechter et al., 2012). To identify novel effectors in *Fusarium* sp., *Stagonospora nodorum*, and *Puccinia graminis* f.sp. *tritici* fungi, Sperschneider et al. performed unsupervised clustering based on 35 sequence-derived features, including amino acid composition (Sperschneider et al., 2013, 2014). Several clusters were characterized by strong biases in amino acid usage, such as the cluster including the three *S. nodorum* effectors SnToxA, SnTox1 and SnTox3 enriched in small and non-polar amino acids and the cluster including *F. oxysporum* f. sp. *lycopersici* SIX3 featuring high average positive protein charge and a significantly higher percentage of Pro, Ser and Thr (Sperschneider et al., 2013). Similarly, secreted effectors of fungi from the *Sclerotiniaceae* family could be enriched in Thr and depleted in Glu and Lys compared to the rest of the proteome. This suggests that amino acid usage bias is a property that may be shared by sets of secreted proteins with unrelated function and from distant pathogen lineages. Consistent with Glu and Lys being disorder-promoting amino acids, we found that secreted proteins of *Sclerotiniaceae* species show lower disorder frequency in hot loops that the rest of the proteome. Effectors of bacterial pathogens were shown to be highly enriched in long disordered regions, presumably to facilitate effector translocation into the host cell, host function mimicry and evasion of the host immune system (Marín et al., 2013). Intrinsic protein disorder was shown to promote high specificity and low affinity protein-ligand interactions (Zhou, 2012; Chu and Wang, 2014). While these properties could be advantageous for host-specific effectors of biotrophic pathogens, for which avoiding detection by the host is critical, opposite requirements may shape the evolution of effectors from broad-range necrotrophic pathogens. Indeed, a relatively low specificity may allow effectors to function during colonization of diverse host species. It is also believed that detection by the host would not be detrimental, and could even be beneficial, to some necrotrophic plant pathogens (Govrin and Levine, 2000). In that case, effectors with high affinity for their targets would not be counter-selected by the host immune system, and would instead favor *Sclerotiniaceae* fungi in the competition with other microbes for plant-derived resources.

Cross species comparative analysis has been successfully applied to the identification of novel and specialized virulence mechanisms on the one hand, and to the identification of optimization principles governing the evolution of proteins under given constraints on the other hand. In nature, *S. borealis* proteins have undergone optimization under specific environmental constraints, including cold, over an irreproducible time at the scale of human life. Comparative genomics approaches therefore have the potential to reveal protein specialization and optimization principles that are not easily accessible through experimental evolution experiments. Indeed, selecting optimized enzyme variants, especially for thermostability, through random mutagenesis often requires exploring a large library of mutants or experimental setups maintaining an appropriate pressure of selection to collect the optimized variants (Kuchner and Arnold, 1997; Lebbink et al., 2000). Comparative genomics can accelerate discoveries usually relying on time consuming screens (Xiao et al., 2008). The biochemical properties of cold-active proteins make them attractive in biochemical, bioremediation, and industrial processes for food, biofuels and pharmaceutical production notably (Cavicchioli et al., 2011). Plant pathogenic fungi in particular present a vast reservoir of biopolymer degrading enzymes adapted to a wide range of temperatures and environments. Functional analyses will be required to test whether the activity of candidates highlighted in this work have applied potential. In the long term, the analysis of optimization principles governing the evolution of secreted proteins from important fungal pathogens may prove useful in improving plant health with the design of crops resistant to broad host range pathogens and to cold stress, and to develop novel strategies for the production of renewable energy relying on the bio-conversion of plant biomass.

## Materials and methods

### Genome sources

We retrieved three predicted proteomes (*Sclerotinia sclerotiorum v1.0, Botrytis cinerea v1.0* and *Sclerotinia borealis F-4157*) from the Joint Genome Institute (http://jgi.doe.gov/) and NCBI (http://www.ncbi.nlm.nih.gov/) in fasta format. As a cautionary note: the proteome sequences that form the basis of our analyses had originally been predicted by various techniques and may thus be of varying quality and completeness. *S. sclerotiorum* gene expression data was obtained from http://urgi.versailles.inra.fr/Data/.

### Gene ontology annotation and enrichment analysis

The Gene Ontology was collected from the Gene Ontology Consortium website (http://geneontology.org/) in obo format. Assignment of the Gene Ontology annotation of the three sets of protein sequences was performed using InterProScan (Jones et al., 2014). GO enrichments analysis was performed using the Biological Networks Gene Ontology plug-in (Maere et al., 2005) in Cytoscape 3.2.1 with the following parameters: a hypergeometric test for statistical analysis with a Bonferroni Family-Wise Error rate correction and a significance level of 0.05.

### Ortholog prediction

Ortholog prediction was performed with standalone InParanoid 4.0 (Ostlund et al., 2010) using all vs. all Basic Local Alignment Search Tool (BLAST) algorithms and the following parameters: the BLOSUM62 matrix, a score cut-off of 50 bits and a minimal sequence overlap area of 0.5 (Altschul et al., 1990; Remm et al., 2001). Two pairwise InParanoid comparisons (*S. borealis* vs. *S. sclerotiorum* and *S. borealis* vs. *B. cinerea*) were ran first on complete proteomes, leading to the identification 6717 COGs, then using only conserved regions of *S. sclerotiorum* proteins (“overlapping regions”) as input (Figure 2). Finally alignments producing a consensus sequence shorter than 200 amino acids were excluded leading to 5531 COGs.

### Pipeline for collecting multiple ortholog alignments

First, ortholog predictions were performed as described in previous section between one organism, called reference organism in the following (*S. sclerotiorum*), and each other organism included in the analysis (*B. cinerea* and *S. borealis*). Only core groups of orthologous proteins harboring one member from each species were retained. Then, the common overlapping sequences in the reference organism to the others organisms were selected according to BLAST begin and end alignment positions. The maximal begin and the minimal end were used to defined the overlapping sequences. Overlapping sequences with lower than 200 amino acids length were excluded. The obtained overlapping sequences in the reference organism were used to run a new round of ortholog prediction with each other organisms. The consensus sequences, or core ortholog groups alignments, in each organisms were selected accordingly to BLAST begin and end alignment positions using the minimal begin and the maximal end obtained through the all orthologs predicted. The consensus sequences with lower than 200 amino acids length were excluded.

### Amino acid and disorder analysis

Protein amino acid usage was assessed by calculating the frequency of each of 20 amino acids in protein sequences. Prediction of disorder probability of protein amino acid was performed with DisEMBL vs. 1.4 computational tool (Linding et al., 2003) on the full length proteins. In case of analysis of a protein sequence subset, like for the core ortholog groups alignments (see previous section), the disorder probability of each amino acid in the subset were taken from the disorder probability of this amino acid in the full length protein. This was done to avoid miss attribution of disorder probability in a subset of a sequence since surrounding of amino acid in the sequence are of importance to calculate its own disorder probability.

### Secretome prediction and protein motif annotation

Analysis by SignalP4.1 was performed at http://www.cbs.dtu.dk using default parameters. Protein localization was predicted with PSORT II software using the WoLF PSORT extension (Horton et al., 2007) for organism type “fungi.” Proteins were defined as part of the secretome when containing both signal peptide and extracellular predicted localization and were excluded if they possess a trans-membrane region predicted by TMHMM (Sonnhammer et al., 1998). Glycosylphosphatidylinositol anchored proteins were identified using Fraganchor (Poisson et al., 2007); N-glycosylation sites were predicted using GlycoEP (Chauhan et al., 2013).

### Statistical analysis and sTEKhot index determination

All statistical tests were computed with R.Studio software. Wilcoxon test was used for significance analysis. Difference was considered significant for *p*-values inferior to 0.05. Significantly enriched or depleted amino acids and disorder frequency in *S. borealis* common set of core ortholog groups' alignments compared to *S. sclerotiorum* and *B. cinerea* core ortholog groups alignments, but found to be not significantly different between *S. sclerotiorum* and *B. cinerea*, were further used for computing the environmental condition adaptation index (sTEKhot). Thr frequency (T_f_) found to be over represented in *S. borealis* were added to the numerator of the index, whereas Lys (K_f_), Glu (E_f_) and hot loops (HotLOOP_f_) frequencies found to be under represented were added to the denominator. Each metrics were normalized by their own median (X_mf_, where X is the considered metric) through the all set of proteome used in the analysis (*S. borealis* plus *S. sclerotiorum* plus *B. cinerea*). This normalization assures similar contribution of each metrics to the index.

(2) $$\begin{array}{l} sTEKhot=\frac{\frac{T_{f}}{T_{mf}}}{\frac{K_{f}}{K_{mf}}+\frac{E_{f}}{E_{mf}}+\frac{{HotLOOP}_{f}}{{HotLOOP}_{mf}}} \end{array}$$

sTEKhot value was calculated for every protein of the three proteomes according to (2). The list of proteins with the top 635 sTEKhot (>1) corresponded exactly to proteins with the top T_f_-(E_f_ + K_f_ + HotLOOP_f_) values supporting the robustness of the arithmetic design of the sTEKhot index in this dataset.

### Random shuffling of sTEKhot

Random sTEKhot indexes were calculated by shuffling amino acid and hotloop frequencies in Equation (2) with any of the observed amino acid and hotloop frequencies for a given organism. The random index is therefore defined by Equation (3) in which W, X, Y, and Z are randomly selected observed frequencies.

(3) $$\begin{array}{l} RANDOMindex=\frac{\frac{X_{f}}{X_{mf}}}{\frac{Y_{f}}{Y_{mf}}+\frac{Z_{f}}{Z_{mf}}+\frac{W_{f}}{W_{mf}}} \end{array}$$

Indexes were calculated separately for the three proteomes and secretomes. Random sTEKhot medians and Wilcoxon ranking test *p*-values were extracted from 300 independent runs.

### Protein structure modeling and analysis

Protein structure modeling was performed with the I-TASSER server (Zhang, 2008) using SS1G_10836 and SS1G_03146 full length sequences as queries. SS1G_10836 best model C-score was -3.22; best TM score was 0.875 (RMSD 2.27Å) with model 4KE2. SS1G_03146 best model C-score was -2.28; best TM score was 0.677 (RMSD 3.07Å) with model 4MAH.

### Gene expression analysis

One-centimeter long leaves were collected and grinded twice for 30 s at maximum frequency in a Retsch MM40 mixer. Total RNA extraction was performed with Macherey-Nagel Nucleospin RNA extraction kit following the manufacturer's instructions. One μg of total RNA was used for cDNA synthesis in a 20-μL reaction according to Roche Transcriptor Reverse Transcriptase protocol, using 0.5 μL of SuperScript II reverse transcriptase (Invitrogen), 1 μg of oligo(dT), and 10 nmol of dNTP. cDNAs (diluted 1:10) were used as templates in the quantitative RT-PCR analysis. Quantitative RT-PCR was performed using gene-specific primers (Table S6) with LightCycler 480 apparatus (Roche Diagnostics). Quantitative PCR reaction was performed using the SYBR GREEN I protocol (5 pmol of each primer and 5 μL of RT reaction product in a 7 μL final reaction volume). The PCR conditions were 9 min at 95°C, followed by 45 cycles of 5 s at 95°C, 10 s at 65°C, and 20 s at 72°C. Expression values of *SS1G_10836* and *SS1G_03146* were normalized based on expression of *SS1G_04652* and *SS1G_12196* housekeeping genes. Values from two biological replicates are shown, error bars show standard error of the mean.

## Author contributions

TB, RP, and SR designed and performed analyses. SR conceived the study. TB, RP, and SR wrote the manuscript.

### Conflict of interest statement

The authors declare that the research was conducted in the absence of any commercial or financial relationships that could be construed as a potential conflict of interest.

## Supplementary material

Figure S1 **Network representation of gene ontologies (GOs) of proteins with sTEKhot >1 in *S. sclerotiorum* proteome**. Nodes correspond to GOs are sized according to the number of proteins with sTEKhot >1. They are colored from yellow to orange according to the *p*-value of a hypergeometric test for enrichment in proteins with sTEKhot >1 compared to whole proteomes.

Figure S2 **Network representation of gene ontologies (GOs) of proteins with sTEKhot >1 in *B. cinerea* proteome**. Nodes correspond to GOs are sized according to the number of proteins with sTEKhot >1. They are colored from yellow to orange according to the *p*-value of a hypergeometric test for enrichment in proteins with sTEKhot >1 compared to whole proteomes.

Figure S3 **Distribution of sTEKhot values for non-secreted proteins and mature secreted proteins (signal peptide removed) in *S. borealis, S. sclerotiorum* and *B. cinerea***.

Figure S4 **Distribution of best BlastP bit scores (log-scaled scores) using *S. borealis* non-secreted proteins and secreted proteins as queries against *S. sclerotiorum* or *B. cinerea* proteomes**. Lower scores for searches using *S. borealis* secretome as query indicate that *S. borealis* secreted proteins are less conserved than non-secreted proteins. *P*-values of a Student *t*-test for differences between non-secreted and secreted proteins are indicated.

## References

[B1] Altschul S. F. Gish W. Miller W. Myers E. W. Lipman D. J. (1990). Basic local alignment search tool. J. Mol. Biol. 215, 403–410. 10.1016/S0022-2836(05)80360-2 2231712

[B2] Amselem J. Cuomo C. A. van Kan J. A. L. Viaud M. Benito E. P. Couloux A.. (2011). Genomic analysis of the necrotrophic fungal pathogens *Sclerotinia sclerotiorum* and *Botrytis cinerea*. PLoS Genet. 7:e1002230. 10.1371/journal.pgen.1002230 21876677 PMC3158057

[B3] Arnold R. Brandmaier S. Kleine F. Tischler P. Heinz E. Behrens S.. (2009). Sequence-based prediction of type III secreted proteins. PLoS Pathog. 5:e1000376. 10.1371/journal.ppat.1000376 19390696 PMC2669295

[B4] Basu K. Graham L. A. Campbell R. L. Davies P. L. (2015). Flies expand the repertoire of protein structures that bind ice. Proc. Natl. Acad. Sci. U.S.A. 112, 737–742. 10.1073/pnas.1422272112 25561557 PMC4311821

[B83] Bolton M. D. Thomma B. P. H. J. Nelson B. D. (2006). *Sclerotinia sclerotiorum* (Lib.) de Bary: biology and molecular traits of a cosmopolitan pathogen. Mol. Plant Pathol. 7, 1–16. 10.1111/j.1364-3703.2005.00316.x 20507424

[B5] Burra P. V. Kalmar L. Tompa P. (2010). Reduction in structural disorder and functional complexity in the thermal adaptation of prokaryotes. PLoS ONE 5:e12069. 10.1371/journal.pone.0012069 20711457 PMC2920320

[B6] Casanueva A. Tuffin M. Cary C. Cowan D. A. (2010). Molecular adaptations to psychrophily: the impact of “omic” technologies. Trends Microbiol. 18, 374–381. 10.1016/j.tim.2010.05.002 20646925

[B7] Catanzariti A.-M. Dodds P. N. Lawrence G. J. Ayliffe M. A. Ellis J. G. (2006). Haustorially expressed secreted proteins from flax rust are highly enriched for avirulence elicitors. Plant Cell 18, 243–256. 10.1105/tpc.105.035980 16326930 PMC1323496

[B8] Cavicchioli R. Amils R. Wagner D. McGenity T. (2011). Life and applications of extremophiles. Environ. Microbiol. 13, 1903–1907. 10.1111/j.1462-2920.2011.02512.x 22236328

[B9] Ceroni A. Passerini A. Vullo A. Frasconi P. (2006). DISULFIND: a disulfide bonding state and cysteine connectivity prediction server. Nucleic Acids Res. 34, W177–W181. 10.1093/nar/gkl266 16844986 PMC1538823

[B10] Chauhan J. S. Rao A. Raghava G. P. S. (2013). *In silico* platform for prediction of N-, O- and C-glycosites in eukaryotic protein sequences. PLoS ONE 8:e67008. 10.1371/journal.pone.0067008 23840574 PMC3695939

[B11] Chu X. Wang J. (2014). Specificity and affinity quantification of flexible recognition from underlying energy landscape topography. PLoS Comput. Biol. 10:e1003782. 10.1371/journal.pcbi.1003782 25144525 PMC4140643

[B12] Dassarma S. Capes M. D. Karan R. Dassarma P. (2013). Amino acid substitutions in cold-adapted proteins from halorubrum lacusprofundi, an extremely halophilic microbe from antarctica. PLoS ONE 8:e58587. 10.1371/journal.pone.0058587 23536799 PMC3594186

[B13] Dean R. Van Kan J. A. L. Pretorius Z. A. Hammond-Kosack K. E. Di Pietro A. Spanu P. D.. (2012). The Top 10 fungal pathogens in molecular plant pathology. Mol. Plant Pathol. 13, 414–430. 10.1111/j.1364-3703.2011.00783.x 22471698 PMC6638784

[B14] Farr D. F. Rossman A. Y. (2015). Fungal Databases. Systematic Mycology and Microbiology Laboratory, ARS, USDA. Available online at: http://nt.ars-grin.gov/fungaldatabases/ (Retrieved March 31, 2015).

[B15] Feller G. (2003). Molecular adaptations to cold in psychrophilic enzymes. Cell. Mol. Life Sci. 60, 648–662. 10.1007/s00018-003-2155-3 12785714 PMC11138853

[B16] Feller G. (2007). Life at low temperatures: is disorder the driving force? Extremophiles 11, 211–216. 10.1007/s00792-006-0050-1 17160345

[B17] Frías M. González C. Brito N. (2011). BcSpl1, a cerato-platanin family protein, contributes to *Botrytis cinerea* virulence and elicits the hypersensitive response in the host. New Phytol. 192, 483–495. 10.1111/j.1469-8137.2011.03802.x 21707620

[B18] Gianese G. Argos P. Pascarella S. (2001). Structural adaptation of enzymes to low temperatures. Protein Eng. 14, 141–148. 10.1093/protein/14.3.141 11342709

[B19] Goodchild A. Saunders N. F. W. Ertan H. Raftery M. Guilhaus M. Curmi P. M. G.. (2004). A proteomic determination of cold adaptation in the *Antarctic archaeon, Methanococcoides burtonii*. Mol. Microbiol. 53, 309–321. 10.1111/j.1365-2958.2004.04130.x 15225324

[B20] Govrin E. M. Levine A. (2000). The hypersensitive response facilitates plant infection by the necrotrophic pathogen *Botrytis* cinerea. Curr. Biol. 10, 751–757. 10898976 10.1016/s0960-9822(00)00560-1

[B21] Guyon K. Balagué C. Roby D. Raffaele S. (2014). Secretome analysis reveals effector candidates associated with broad host range necrotrophy in the fungal plant pathogen *Sclerotinia sclerotiorum*. BMC Genomics 15:336. 10.1186/1471-2164-15-336 24886033 PMC4039746

[B22] Hacquard S. Joly D. L. Lin Y.-C. Tisserant E. Feau N. Delaruelle C.. (2012). A comprehensive analysis of genes encoding small secreted proteins identifies candidate effectors in Melampsora larici-populina (poplar leaf rust). Mol. Plant. Microbe. Interact. 25, 279–293. 10.1094/MPMI-09-11-0238 22046958

[B23] He X. Han K. Hu J. Yan H. Yang J.-Y. Shen H.-B.. (2015). TargetFreeze: identifying antifreeze proteins via a combination of weights using sequence evolutionary information and pseudo amino acid composition. J. Membr. Biol. 10.1007/s00232-015-9811-z. [Epub ahead of print]. 26058944

[B24] Heard S. Brown N. A. Hammond-Kosack K. (2015). An interspecies comparative analysis of the predicted secretomes of the necrotrophic plant pathogens *Sclerotinia sclerotiorum* and *Botrytis cinerea*. PLoS One 10:e0130534. 10.1371/journal.pone.0130534 26107498 PMC4480369

[B25] Hemsworth G. R. Henrissat B. Davies G. J. Walton P. H. (2014). Discovery and characterization of a new family of lytic polysaccharide monooxygenases. Nat. Chem. Biol. 10, 122–126. 10.1038/nchembio.1417 24362702 PMC4274766

[B26] Horton P. Park K.-J. Obayashi T. Fujita N. Harada H. Adams-Collier C. J.. (2007). WoLF PSORT: protein localization predictor. Nucleic Acids Res. 35, W585–W587. 10.1093/nar/gkm259 17517783 PMC1933216

[B27] Hoshino T. Terami F. Tkachenko O. B. Tojo M. Matsumoto N. (2010). Mycelial growth of the snow mold fungus, *Sclerotinia borealis*, improved at low water potentials: an adaption to frozen environment. Mycoscience 51, 98–103. 10.1007/S10267-009-0013-3

[B28] Hu X. Xiao G. Zheng P. Shang Y. Su Y. Zhang X.. (2014). Trajectory and genomic determinants of fungal-pathogen speciation and host adaptation. Proc. Natl. Acad. Sci. U.S.A. 111, 16796–16801. 10.1073/pnas.1412662111 25368161 PMC4250126

[B29] Jones J. D. G. Dangl J. L. (2006). The plant immune system. Nature 444, 323–329. 10.1038/nature05286 17108957

[B30] Jones P. Binns D. Chang H.-Y. Fraser M. Li W. McAnulla C.. (2014). InterProScan 5: genome-scale protein function classification. Bioinformatics 30, 1236–1240. 10.1093/bioinformatics/btu031 24451626 PMC3998142

[B31] Judet-Correia D. Bollaert S. Duquenne A. Charpentier C. Bensoussan M. Dantigny P. (2010). Validation of a predictive model for the growth of *Botrytis cinerea* and *Penicillium expansum* on grape berries. Int. J. Food Microbiol. 142, 106–113. 10.1016/j.ijfoodmicro.2010.06.009 20619474

[B32] Juenger T. Bergelson J. (1998). Pairwise versus diffuse natural selection and the multiple herbivores of scarlet gilia, Ipomopsis aggregata. Evolution 52, 1583–1592. 10.2307/2411332 28565335

[B33] Julenius K. Pedersen A. G. (2006). Protein evolution is faster outside the cell. Mol. Biol. Evol. 23, 2039–2048. 10.1093/molbev/msl081 16891379

[B34] Kondo H. Hanada Y. Sugimoto H. Hoshino T. Garnham C. P. Davies P. L.. (2012). Ice-binding site of snow mold fungus antifreeze protein deviates from structural regularity and high conservation. Proc. Natl. Acad. Sci. U.S.A. 109, 9360–9365. 10.1073/pnas.1121607109 22645341 PMC3386094

[B35] Kubicek C. P. Starr T. L. Glass N. L. (2014). Plant cell wall-degrading enzymes and their secretion in plant-pathogenic fungi. Annu. Rev. Phytopathol. 52, 427–451. 10.1146/annurev-phyto-102313-045831 25001456

[B36] Kuchner O. Arnold F. H. (1997). Directed evolution of enzyme catalysts. Trends Biotechnol. 15, 523–530. 10.1016/S0167-7799(97)01138-4 9418307

[B37] Lebbink J. H. Kaper T. Bron P. van der Oost J. de Vos W. M. (2000). Improving low-temperature catalysis in the hyperthermostable Pyrococcus furiosus beta-glucosidase CelB by directed evolution. Biochemistry 39, 3656–3665. 10.1021/bi991483q 10736164

[B38] Liao B.-Y. Weng M.-P. Zhang J. (2010). Impact of extracellularity on the evolutionary rate of mammalian proteins. Genome Biol. Evol. 2, 39–43. 10.1093/gbe/evp058 20333223 PMC2839354

[B39] Linding R. Jensen L. J. Diella F. Bork P. Gibson T. J. Russell R. B. (2003). Protein disorder prediction: implications for structural proteomics. Structure 11, 1453–1459. 10.1016/j.str.2003.10.002 14604535

[B40] Liu T. Song T. Zhang X. Yuan H. Su L. Li W.. (2014). Unconventionally secreted effectors of two filamentous pathogens target plant salicylate biosynthesis. Nat. Commun. 5, 4686. 10.1038/ncomms5686 25156390 PMC4348438

[B41] Maere S. Heymans K. Kuiper M. (2005). BiNGO: a Cytoscape plugin to assess overrepresentation of gene ontology categories in biological networks. Bioinformatics 21, 3448–3449. 10.1093/bioinformatics/bti551 15972284

[B42] Mardanov A. V. Beletsky A. V. Kadnikov V. V. Ignatov A. N. Ravin N. V. (2014a). Draft genome sequence of *Sclerotinia borealis*, a psychrophilic plant pathogenic fungus. Genome Announc. 2:e01175-13. 10.1128/genomea.01175-13 24459262 PMC3900894

[B43] Mardanov A. V. Beletsky A. V. Kadnikov V. V. Ignatov A. N. Ravin N. V. (2014b). The 203 kbp mitochondrial genome of the phytopathogenic fungus *Sclerotinia borealis* reveals multiple invasions of introns and genomic duplications. PLoS ONE 9:e107536. 10.1371/journal.pone.0107536 25216190 PMC4162613

[B44] Marín M. Uversky V. N. Ott T. (2013). Intrinsic disorder in pathogen effectors: protein flexibility as an evolutionary hallmark in a molecular arms race. Plant Cell 25, 3153–3157. 10.1105/tpc.113.116319 24038649 PMC3809524

[B45] Mayans O. Scott M. Connerton I. Gravesen T. Benen J. Visser J.. (1997). Two crystal structures of pectin lyase A from Aspergillus reveal a pH driven conformational change and striking divergence in the substrate-binding clefts of pectin and pectate lyases. Structure 5, 677–689. 10.1016/S0969-2126(97)00222-0 9195887

[B46] McDermott J. E. Corrigan A. Peterson E. Oehmen C. Niemann G. Cambronne E. D.. (2011). Computational prediction of type III and IV secreted effectors in gram-negative bacteria. Infect. Immun. 79, 23–32. 10.1128/IAI.00537-10 20974833 PMC3019878

[B47] Methé B. A. Nelson K. E. Deming J. W. Momen B. Melamud E. Zhang X.. (2005). The psychrophilic lifestyle as revealed by the genome sequence of Colwellia psychrerythraea 34H through genomic and proteomic analyses. Proc. Natl. Acad. Sci. U.S.A. 102, 10913–10918. 10.1073/pnas.0504766102 16043709 PMC1180510

[B48] Metpally R. P. R. Reddy B. V. B. (2009). Comparative proteome analysis of psychrophilic versus mesophilic bacterial species: insights into the molecular basis of cold adaptation of proteins. BMC Genomics 10:11. 10.1186/1471-2164-10-11 19133128 PMC2653534

[B49] Meyer D. F. Noroy C. Moumène A. Raffaele S. Albina E. Vachiéry N. (2013). Searching algorithm for type IV secretion system effectors 1.0: a tool for predicting type IV effectors and exploring their genomic context. Nucleic Acids Res. 41, 9218–9229. 10.1093/nar/gkt718 23945940 PMC3814349

[B50] Möbius N. Hertweck C. (2009). Fungal phytotoxins as mediators of virulence. Curr. Opin. Plant Biol. 12, 390–398. 10.1016/j.pbi.2009.06.004 19608453

[B51] Oliva R. F. Cano L. M. Raffaele S. Win J. Bozkurt T. O. Belhaj K.. (2015). A recent expansion of the RXLR effector gene Avrblb2 is maintained in global populations of Phytophthora infestans indicating different contributions to virulence. Mol. Plant. Microbe. Interact. 28, 901–912. 10.1094/MPMI-12-14-0393-R 25894205

[B52] Ostlund G. Schmitt T. Forslund K. Köstler T. Messina D. N. Roopra S.. (2010). InParanoid 7: new algorithms and tools for eukaryotic orthology analysis. Nucleic Acids Res. 38, D196–D203. 10.1093/nar/gkp931 19892828 PMC2808972

[B53] Persoons A. Morin E. Delaruelle C. Payen T. Halkett F. Frey P.. (2014). Patterns of genomic variation in the poplar rust fungus Melampsora larici-populina identify pathogenesis-related factors. Front. Plant Sci. 5:450. 10.3389/fpls.2014.00450 25309551 PMC4164029

[B54] Poisson G. Chauve C. Chen X. Bergeron A. (2007). FragAnchor: a large-scale predictor of glycosylphosphatidylinositol anchors in eukaryote protein sequences by qualitative scoring. Genomics Proteomics Bioinformatics 5, 121–130. 10.1016/S1672-0229(07)60022-9 17893077 PMC5054108

[B55] Raffaele S. Farrer R. A. Cano L. M. Studholme D. J. MacLean D. Thines M.. (2010). Genome evolution following host jumps in the Irish potato famine pathogen lineage. Science 330, 1540–1543. 10.1126/science.1193070 21148391

[B56] Rech G. E. Sanz-Martín J. M. Anisimova M. Sukno S. A. Thon M. R. (2014). Natural selection on coding and noncoding DNA sequences is associated with virulence genes in a plant pathogenic fungus. Genome Biol. Evol. 6, 2368–2379. 10.1093/gbe/evu192 25193312 PMC4202328

[B57] Remm M. Storm C. E. Sonnhammer E. L. (2001). Automatic clustering of orthologs and in-paralogs from pairwise species comparisons. J. Mol. Biol. 314, 1041–1052. 10.1006/jmbi.2000.5197 11743721

[B58] Roux F. Voisin D. Badet T. Balagué C. Barlet X. Huard-Chauveau C.. (2014). Resistance to phytopathogens e tutti quanti: placing plant quantitative disease resistance on the map. Mol. Plant Pathol. 15, 427–432. 10.1111/mpp.12138 24796392 PMC6638617

[B59] Saunders D. G. O. Win J. Cano L. M. Szabo L. J. Kamoun S. Raffaele S. (2012). Using hierarchical clustering of secreted protein families to classify and rank candidate effectors of rust fungi. PLoS ONE 7:e29847. 10.1371/journal.pone.0029847 22238666 PMC3253089

[B60] Schardl C. L. Young C. A. Hesse U. Amyotte S. G. Andreeva K. Calie P. J.. (2013). Plant-symbiotic fungi as chemical engineers: multi-genome analysis of the clavicipitaceae reveals dynamics of alkaloid loci. PLoS Genet. 9:e1003323. 10.1371/journal.pgen.1003323 23468653 PMC3585121

[B61] Schechter L. M. Valenta J. C. Schneider D. J. Collmer A. Sakk E. (2012). Functional and computational analysis of amino acid patterns predictive of type III secretion system substrates in Pseudomonas syringae. PLoS ONE 7:e36038. 10.1371/journal.pone.0036038 22558318 PMC3338616

[B62] Schneider E. F. Seaman W. L. (1987). Snow mold diseases and their distribution on winter wheat in Ontario in 1982-84. Can. Plant Dis. Surv. 67, 35–39.

[B63] Schornack S. Huitema E. Cano L. M. Bozkurt T. O. Oliva R. Van Damme M.. (2009). Ten things to know about oomycete effectors. Mol. Plant Pathol. 10, 795–803. 10.1111/j.1364-3703.2009.00593.x 19849785 PMC6640533

[B64] Smith D. R. Chapman M. R. (2010). Economical evolution: microbes reduce the synthetic cost of extracellular proteins. MBio 1, 28–32. 10.1128/mBio.00131-10 20824102 PMC2932507

[B65] Sonnhammer E. L. von Heijne G. Krogh A. (1998). A hidden Markov model for predicting transmembrane helices in protein sequences. Proc. Int. Conf. Intell. Syst. Mol. Biol. 6, 175–182. 9783223

[B66] Sperschneider J. Dodds P. N. Gardiner D. M. Manners J. M. Singh K. B. Taylor J. M. (2015). Advances and challenges in computational prediction of effectors from plant pathogenic fungi. PLoS Pathog. 11:e1004806. 10.1371/journal.ppat.1004806 26020524 PMC4447458

[B67] Sperschneider J. Gardiner D. M. Taylor J. M. Hane J. K. Singh K. B. Manners J. M. (2013). A comparative hidden Markov model analysis pipeline identifies proteins characteristic of cereal-infecting fungi. BMC Genomics 14:807. 10.1186/1471-2164-14-807 24252298 PMC3914424

[B68] Sperschneider J. Ying H. Dodds P. N. Gardiner D. M. Upadhyaya N. M. Singh K. B.. (2014). Diversifying selection in the wheat stem rust fungus acts predominantly on pathogen-associated gene families and reveals candidate effectors. Front. Plant Sci. 5:372. 10.3389/fpls.2014.00372 25225496 PMC4150398

[B69] Suhre K. Claverie J.-M. (2003). Genomic correlates of hyperthermostability, an update. J. Biol. Chem. 278, 17198–17202. 10.1074/jbc.M301327200 12600994

[B70] Sun T. Lin F.-H. Campbell R. L. Allingham J. S. Davies P. L. (2014). An antifreeze protein folds with an interior network of more than 400 semi-clathrate waters. Science 343, 795–798. 10.1126/science.1247407 24531972

[B71] Tantos A. Friedrich P. Tompa P. (2009). Cold stability of intrinsically disordered proteins. FEBS Lett. 583, 465–469. 10.1016/j.febslet.2008.12.054 19121309

[B72] Van Noort V. Bradatsch B. Arumugam M. Amlacher S. Bange G. Creevey C.. (2013). Consistent mutational paths predict eukaryotic thermostability. BMC Evol. Biol. 13:7. 10.1186/1471-2148-13-7 23305080 PMC3546890

[B73] Wang G.-Z. Lercher M. J. (2010). Amino acid composition in endothermic vertebrates is biased in the same direction as in thermophilic prokaryotes. BMC Evol. Biol. 10:263. 10.1186/1471-2148-10-263 20807394 PMC2939578

[B74] Wang Y. Wei X. Bao H. Liu S.-L. (2014). Prediction of bacterial type IV secreted effectors by C-terminal features. BMC Genomics 15:50. 10.1186/1471-2164-15-50 24447430 PMC3915618

[B75] Weiberg A. Wang M. Lin F.-M. Zhao H. Zhang Z. Kaloshian I.. (2013). Fungal small RNAs suppress plant immunity by hijacking host RNA interference pathways. Science 342, 118–123. 10.1126/science.1239705 24092744 PMC4096153

[B76] Wicker T. Oberhaensli S. Parlange F. Buchmann J. P. Shatalina M. Roffler S.. (2013). The wheat powdery mildew genome shows the unique evolution of an obligate biotroph. Nat. Genet. 45, 1092–1096. 10.1038/ng.2704 23852167

[B77] Wu B. M. Subbarao K. V. Qin Q. M. (2008). Nonlinear colony extension of *Sclerotinia* minor and *S. sclerotiorum*. Mycologia 100, 902–910. 10.3852/08-021 19202844

[B78] Xiao Z. Bergeron H. Grosse S. Beauchemin M. Garron M.-L. Shaya D.. (2008). Improvement of the thermostability and activity of a pectate lyase by single amino acid substitutions, using a strategy based on melting-temperature-guided sequence alignment. Appl. Environ. Microbiol. 74, 1183–1189. 10.1128/AEM.02220-07 18156340 PMC2258563

[B79] Zeldovich K. B. Berezovsky I. N. Shakhnovich E. I. (2007). Protein and DNA sequence determinants of thermophilic adaptation. PLoS Comput. Biol. 3:e5. 10.1371/journal.pcbi.0030005 17222055 PMC1769408

[B80] Zhang Y. (2008). I-TASSER server for protein 3D structure prediction. BMC Bioinformatics 9:40. 10.1186/1471-2105-9-40 18215316 PMC2245901

[B81] Zhou H.-X. (2012). Intrinsic disorder: signaling via highly specific but short-lived association. Trends Biochem. Sci. 37, 43–48. 10.1016/j.tibs.2011.11.002 22154231 PMC3278522

[B82] Zou L. Nan C. Hu F. (2013). Accurate prediction of bacterial type IV secreted effectors using amino acid composition and PSSM profiles. Bioinformatics 29, 3135–3142. 10.1093/bioinformatics/btt554 24064423 PMC5994942

