# Supplementary figures and images for: Common protein sequence signatures associate with Sclerotinia borealis lifestyle and secretion in fungal pathogens of the Sclerotiniaceae
Source: Front Plant Sci. 2015 Sep 24;6:776. doi: 10.3389/fpls.2015.00776 (PMC4585107; doi:10.3389/fpls.2015.00776)

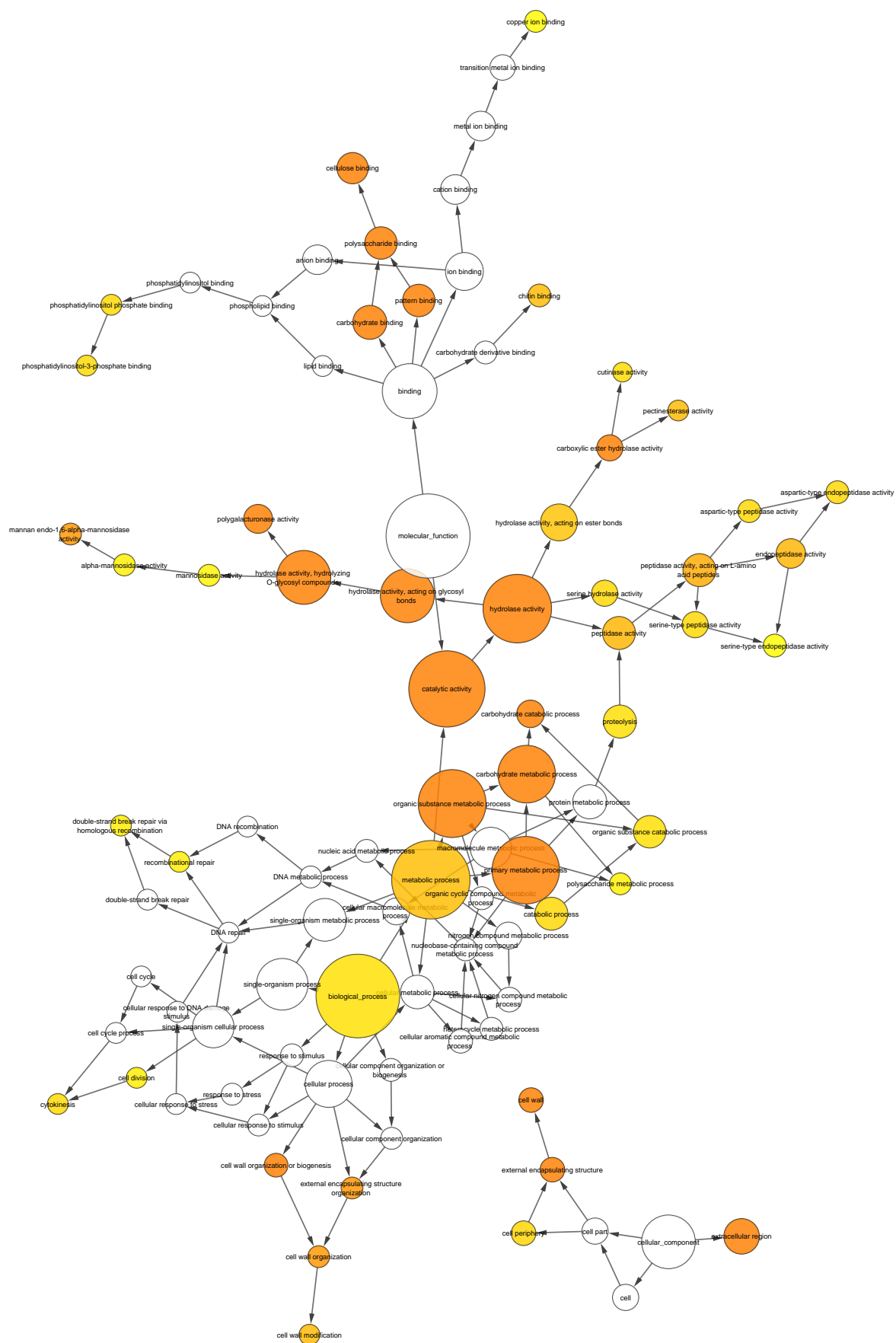

Supplement: Figure S1 — Network representation of gene ontologies (GOs) of proteins with sTEKhot >1 in S. sclerotiorum proteome. Nodes correspond to GOs are sized according to the number of proteins with sTEKhot >1. They are colored from yellow to orange according to the p-value of a hypergeometric test for enrichment in proteins with sTEKhot >1 compared to whole proteomes. [file Image1.PDF]

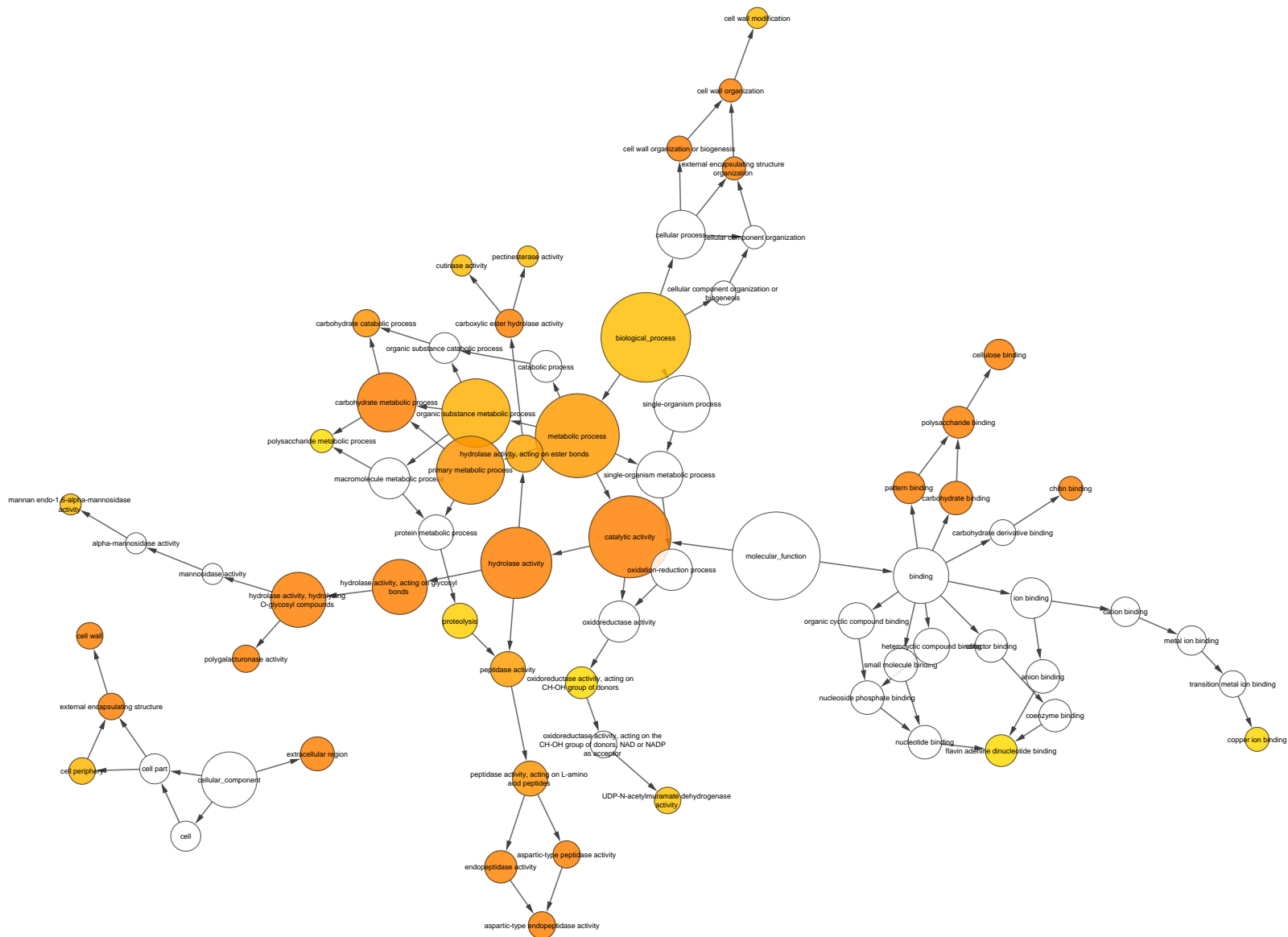

Supplement: Figure S2 — Network representation of gene ontologies (GOs) of proteins with sTEKhot >1 in B. cinerea proteome. Nodes correspond to GOs are sized according to the number of proteins with sTEKhot >1. They are colored from yellow to orange according to the p-value of a hypergeometric test for enrichment in proteins with sTEKhot >1 compared to whole proteomes. [file Image2.PDF]

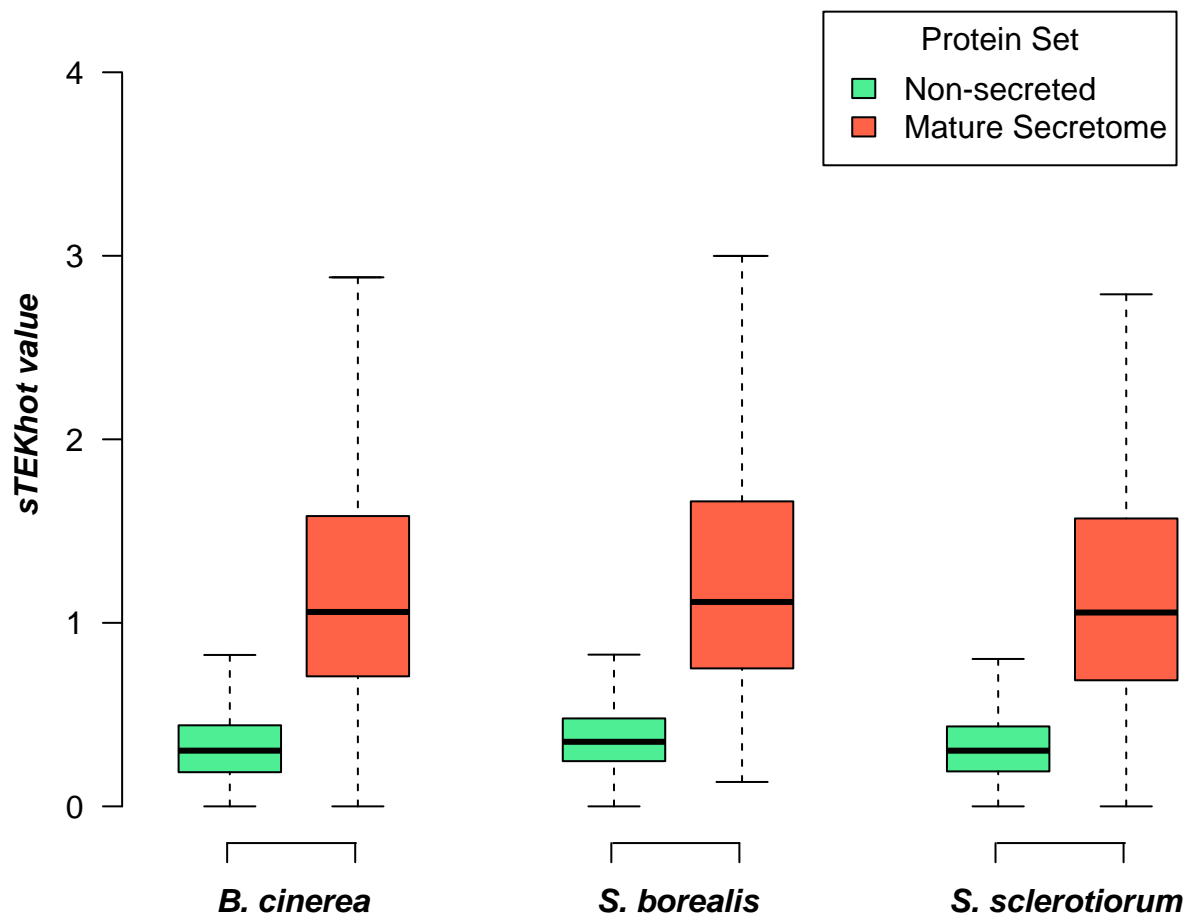

Supplement: Figure S3 — Distribution of sTEKhot values for non-secreted proteins and mature secreted proteins (signal peptide removed) in S. borealis, S. sclerotiorum and B. cinerea. [file Image3.PDF]

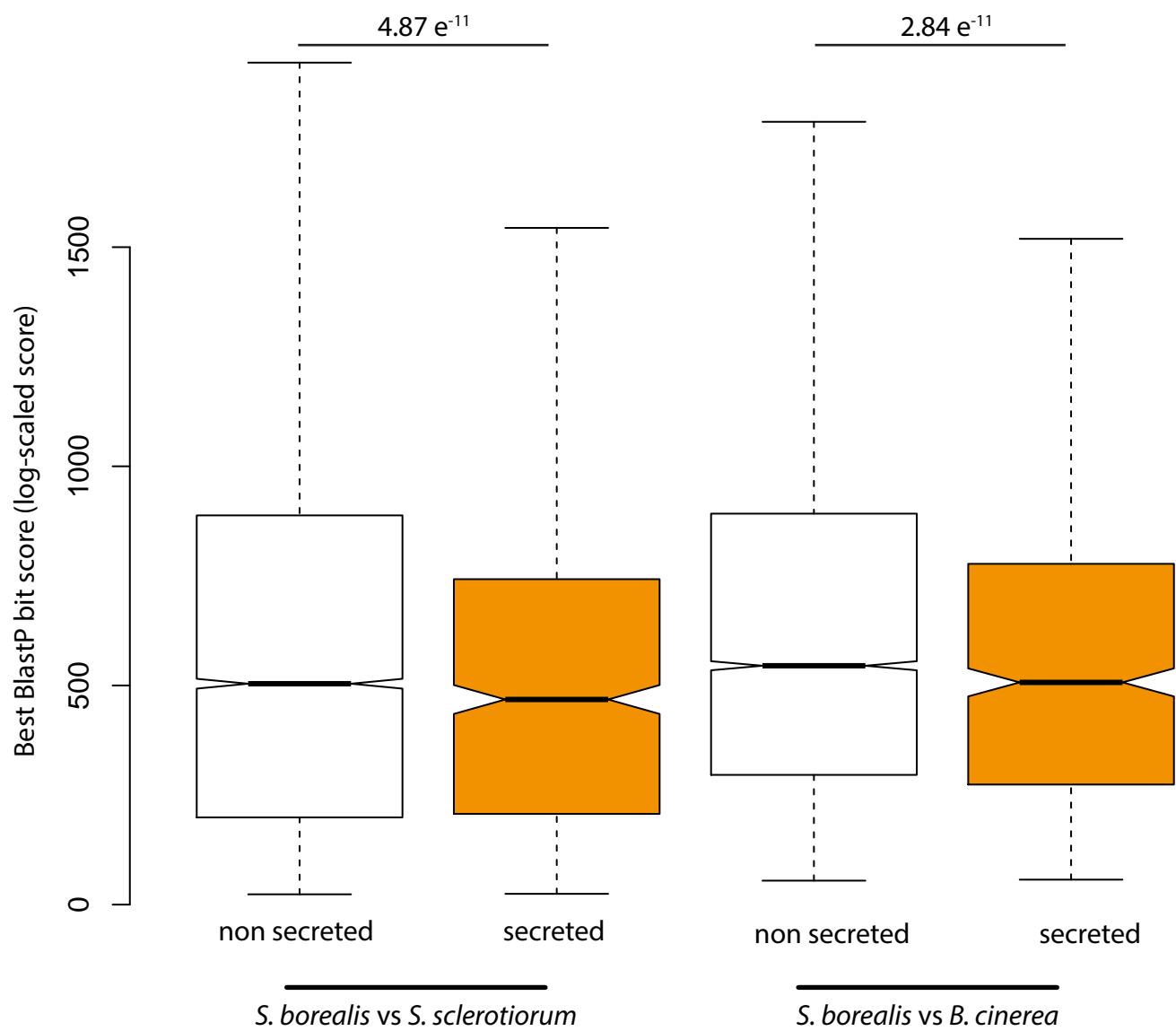

Supplement: Figure S4 — Distribution of best BlastP bit scores (log-scaled scores) using S. borealis non-secreted proteins and secreted proteins as queries against S. sclerotiorum or B. cinerea proteomes. Lower scores for searches using S. borealis secretome as query indicate that S. borealis secreted proteins are less conserved than non-secreted proteins. P-values of a Student t-test for differences between non-secreted and secreted proteins are indicated. [file Image4.PDF]
